# Multi-strategy cooperative scheduling for airport specialized vehicles based on digital twins

**DOI:** 10.1038/s41598-024-66350-0

**Published:** 2024-07-05

**Authors:** Qian Luo, Huaiming Liu, Chang Liu, Qiangqiang Deng

**Affiliations:** 1 College of Computer and Software Engineering, Xihua University, Chengdu, 610039 Sichuan China; 2 Second Research Institute, Civil Aviation Administration of China, Chengdu, 610041 Sichuan China

**Keywords:** Engineering, Aerospace engineering, Electrical and electronic engineering

## Abstract

Efficient specialized vehicle cooperative scheduling is significant for airport operations, particularly during times of high traffic, which reduces the risk of flight delays and increases customer satisfaction. In this paper,we construct a multi-type vehicles collaborative scheduling model with the objectives of minimizing vehicle travel distance and vehicle waiting time. Additionally, a three-layer genetic algorithm is designed, and the crossover and mutation operations are enhanced to address the scheduling model. Due to the numerous uncertainties and stochastic interferences in airport operations, conventional scheduling methods unable to effectively address these challenges, this paper combines improved genetic algorithm, simulation algorithm, and digital twins technology, proposing a multi-strategy scheduling framework for specialized vehicles based on digital twins. The scheduling framework utilises digital twins to capture dynamic data from the airport and continuously adjusts the scheduling plan through the scheduling strategy to ensure robust scheduling for specialized vehicles. In the event of severe delays at the airport, fast and efficient re-scheduling can be achieved. Finally, the effectiveness of the proposed scheduling framework is validated using domestic flight data, and extensive experiments and analyses are conducted in different scenarios. This research contributes to addressing the optimization problem of cooperative scheduling for multi-type vehicles at airports.

## Introduction

**Figure 1:**
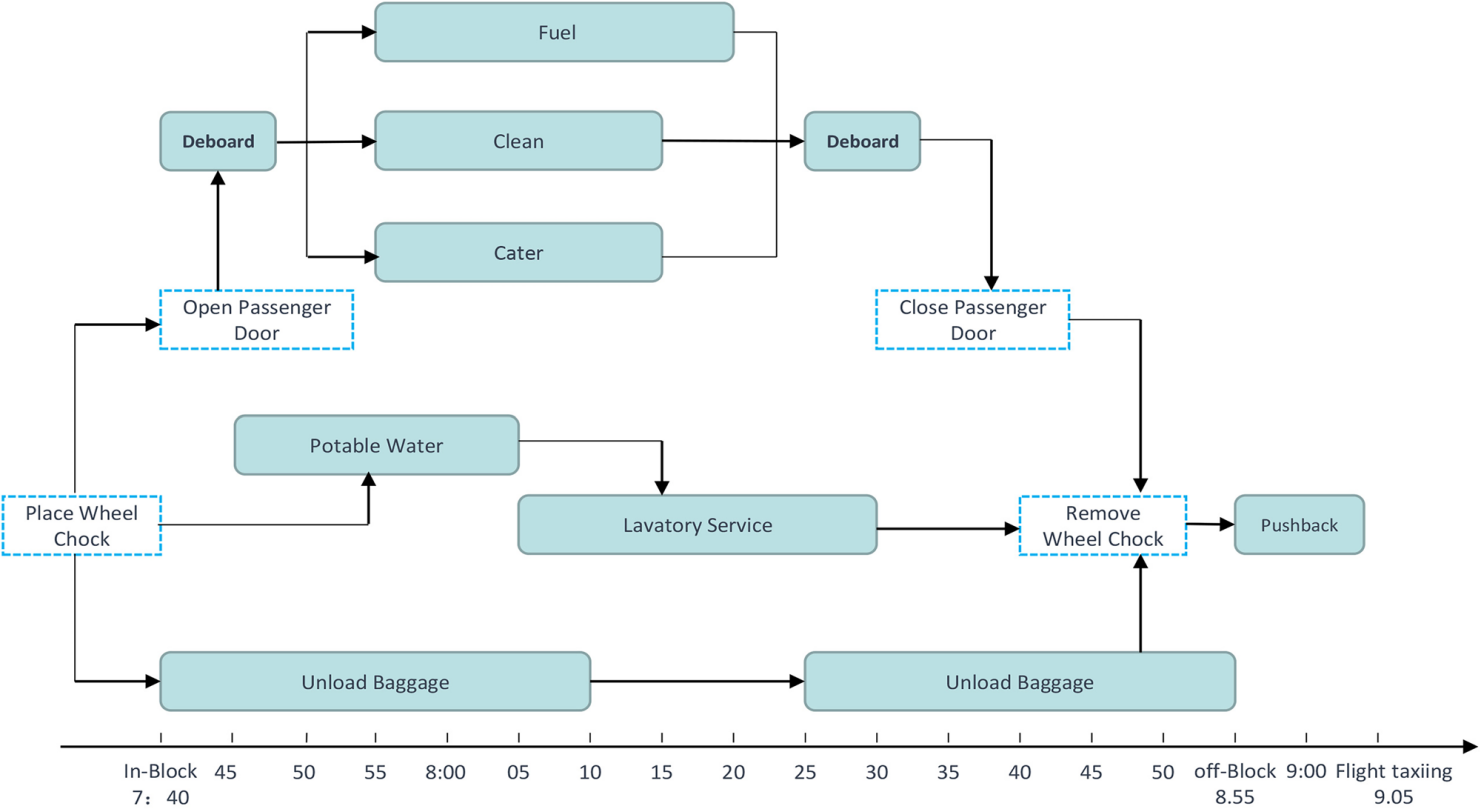
Flight ground-handling process.

In recent years, with the rapid growth of the global civil aviation industry and a significant increase in passenger demand, many international airports are facing the dual challenges of rising air traffic volume and low service efficiency. In airport operations, flight ground-handling services play a crucial role, involving tasks such as aircraft guidance, loading and unloading of baggage and cargo, refueling, water supply, aircraft cleaning, and passenger services^1^,as shown in Fig. 1. These tasks require the use of different specialized vehicles, such as tractors, baggage trucks, fuel trucks, and cleaning trucks. Moreover, these vehicles must complete their services within strict time windows to ensure the punctuality and effectiveness of flight services. According to statistics, apart from weather factors, low utilization of service fleets, poor coordination, and inefficient scheduling are considered the major reasons for flight delays^2^. Particularly for major airports in hub cities, efficient scheduling of specialized vehicles during peak flight times is crucial. However, with the growth in the number of flights, traditional specialized vehicle scheduling strategies are no longer sufficient to meet the high-quality service requirements of airports. Therefore, optimizing the scheduling of specialized vehicles holds significant research importance for reducing flight delays and enhancing service quality. This paper will focus on how to optimize the scheduling of airport specialized vehicles to cope with the increasing number of flights and service demands, thereby enhancing the overall operational efficiency of airports.

In the past few decades, most scholars have focused on optimizing the scheduling of single-type vehicles, such as fuel trucks, ferry vehicles, baggage trucks, and de-icing trucks. Only a few scholars have studied the collaborative scheduling among multi-type vehicles. Currently, most vehicle scheduling problems are modeled as Vehicle Routing Problems with Time Windows (VRPTW), and various methods including linear programming, heuristic algorithms, and machine learning algorithms^3^ are employed to find solutions under different constraint conditions. However, the aforementioned methods typically assume the flight ground-handling process to be a deterministic system, assuming no fluctuations in the flight schedule, fixed service times, and equal travel times for vehicles, thus overlooking the fact that the ground service process is a dynamic system with changing time, space, and demand constraints. Therefore, scheduling solutions computed in deterministic environments cannot meet actual operational requirements.

To address the issue of poor reliability of solutions in uncertain environments, some scholars have modeled the uncertainty of flight departure and arrival times, as well as the fluctuation of ground service times, as parameters in scheduling models^4^, thereby improving the reliability of solutions. However, considering that the flight ground-handling process is inherently a stochastic system, describing uncertainty through parameters is far from sufficient. Therefore, some scholars have recognized the powerful analytical capabilities of simulation technology in stochastic systems, which can reproduce the actual operational processes of airports through simulation models to analyze, validate, and optimize. However, solely using simulation technology for solving can lead to an explosion of the solution search space, making it impractical to compute the optimal solution within a reasonable time^5^. To overcome this challenge, some scholars have integrated optimization algorithms with simulation technology^6^. This method combines simulation, which mimics real-world processes to predict outcomes, with optimization algorithms that seek the best possible solution under given constraints. Optimization algorithms are utilized to compute a set of suboptimal or optimal solutions within the search space, while simulation technology is employed to evaluate various scheduling scenarios. Evaluation metrics from the simulation can then be fed back to the optimization algorithm to enhance its exploration capability within the search space. Solutions obtained through simulation-optimization methods are more reliable in real-world scenarios. However, previous research has predominantly focused on using information from a specific moment as model input, with little utilization of historical and real-time data. This limitation poses challenges for anomaly detection and dynamic scheduling.

In recent years, to achieve precise monitoring and rapid response to random disturbances in the production scheduling process, some scholars have integrated digital twins (DT) technology with simulation-optimization in scheduling optimization problems^7^. DT is a virtual representation of a physical system, used to simulate, analyze, and optimize real-world operations. The airport operating environment is complex and dynamic, making resource allocation and equipment scheduling particularly challenging. The flight ground-handling process involves the coordinated operation of personnel and various equipment. DT can map the dynamic operation process of ground handling services into a virtual space, thereby optimizing scheduling decisions by analyzing real-time states. Specialized vehicles are the primary equipment for ground services, performing tasks assigned by the scheduling system. During operation, issues such as breakdowns, conflicts, and flight delays may arise, affecting the efficiency and scheduling plans of the vehicles. Therefore, a DT-based scheduling system can assist decision-makers in better managing and scheduling personnel and equipment, enabling specialized vehicles to make more informed decisions in dynamic environments.

Overall, both popular simulation-optimization frameworks and DT-based frameworks have limitations in dynamically adjusting and assessing the feasibility of solutions. Firstly, in simulation-optimization frameworks, insufficient modeling depth of the scheduling process and delays in information input hinder optimization algorithms from finding the optimal solution for the current state. As a result, the evaluation outcomes of the simulation models become unreliable. Particularly during emergencies, the inability to adjust solutions dynamically leads to their direct failure. In DT-based frameworks, although data modeling and management improve the efficiency of flight turnaround events^8^, these frameworks primarily offer monitoring functions rather than comprehensive analysis and optimization of the entire airport operation. They tend to provide reactive scheduling in response to emergencies rather than proactive scheduling, failing to achieve iterative optimization throughout the entire operation process.

To address the above issues, we propose a multi-strategy scheduling framework for specialized vehicles based on digital twins. Firstly, the virtual space of the DT continuously updates with the scheduling process, collecting real-time and historical data to provide data support for optimization algorithms and simulation models. This comprehensive consideration of a broader range of information enables more accurate decision-making in the scheduling process. Furthermore, the simulation models in the virtual space of the DT serve as dynamic references for validating scheduling plans, providing fitness feedback to optimization algorithms. This enhances the exploration capability of optimization algorithms in finding optimal solutions. Finally, with the support of DT technology, through information exchange among the physical airport, virtual airport, and scheduling center, iterative optimization of the scheduling process is achieved. This enables rapid responses to unforeseen events during the scheduling process. The main contributions of this paper are summarized as follows:

- We analyze the characteristics of flight ground-handling services, establishing a multi-objective optimization model to minimize travel distance and vehicle waiting time in the scheduling plan, while ensuring low delays.
- We introduce a digital twins-based multi-strategy scheduling framework, enhance the crossover and mutation operations of the genetic algorithm to improve the stability and diversity of the algorithm’s search process. The framework utilizes a simulation program to provide fitness feedback for the optimization algorithm, guiding the search towards more robust solutions.
- We propose an event-driven real-time rescheduling strategy that integrates simulation and optimization modules for rapid solution generation to address dynamic events. The strategy involves repairing and evaluating solutions to minimize delay propagation.

The structure of this paper is as follows. In Section “Literature review” , we provide a literature review on flight ground-handling, uncertainty handling methods, and digital twins technology . Section “Model construction” describes the airport multi-type vehicle scheduling problem and establishes the corresponding scheduling model. Section “Multi-strategy scheduling framework for specialized vehicles based on digital twins” details the proposed framework, including the framework’s operation mechanism and components. It focuses on the data processing module, simulation module, and algorithm module, as well as the specifics of the improved genetic algorithm within the algorithm module. Section “Case study” validates the performance of the proposed improved genetic algorithm through computational experiments and demonstrates the advantages of our approach in different scenarios. Finally, Section “Conclusion” summarizes the research findings and discusses future research directions.

## Literature review

### Flight ground-handling

Over the past few decades, most studies have formulated the scheduling of airport specialized vehicles as a vehicle routing problem with time windows. Based on the number of vehicles, these problems can be categorized into single-type vehicle scheduling and collaborative scheduling of multi-type vehicles. Single-type vehicle scheduling problems include the scheduling of tractor, ferry vehicle, baggage truck, and fuel truck. For instance, Wang proposed a distributed strategy for tractor services, developed an improved NSGA-II algorithm, and successfully reduced delay times^9^. Regarding the scheduling of ferry vehicle, Han introduced a dual-objective mixed-integer linear programming model and designed three heuristic algorithms to enhance efficiency and balance utilization^10^. Similarly, Lv defined the scheduling problem of airport ferry vehicles as an Unrelated Parallel Machine Scheduling (UPMS) problem, introduced a Variable Neighborhood Search (VNS) algorithm and designed five neighborhood structures^11^. Guo addressed the airport baggage truck transportation problem by proposing a genetic algorithm that enhances population diversity and verified the feasibility of the algorithm through simulation with actual data^12^. Likewise, Wang focused on the scheduling of airport fuel trucks and employed a particle swarm optimization algorithm for solving the problem^13^. However, single-type vehicle scheduling often overlooks the diverse nature, time constraints, and interdependencies inherent in flight ground handling, deviating from the actual operational requirements of airports.

Some scholars have conducted research on collaborative scheduling of multi-type vehicles, Padrón proposed an approach to address the correlated routing problem for multi-type vehicles from a global perspective, using a quick heuristic method to explore the solution space^14^. Zhang investigated the scheduling problem of airport vehicles under resource constraints, adjusted vehicles and their procedures through a sequential sorting method, and employed a multi-layer encoding genetic algorithm for solving the problem^15^. Padrón also considered the correlation in the multi-type vehicles collaborative scheduling problem, modeled the constraints of the service process, and maintained consistency through a constraint propagation mechanism^16^. Zhou introduced a Mixed-Integer Linear Programming (MILP) model to address complex routing problems and employed an integrated Large Neighborhood Search (LNS) method with imitation learning and Graph Convolutional Networks (GCN) for solving^17^. Liu considered the service constraints among vehicles and the limitations in the number of vehicles, simplified the multi-type vehicles scheduling problem into sequential and parallel service relationships, and used NSGA-II for solving^18^. Although these studies addressed some mainline services, they lacked a global perspective on coordinating cabin services, energy supply, and baggage loading. There was a lack of in-depth exploration and modeling of the flight ground-handling process, where delays in single service could trigger delays in other services, ultimately resulting in flight delays.

The studies mentioned above all treat the flight ground-handling process as deterministic, ignoring the uncertainties in ground service times for different types of vehicles during actual operations. Additionally, they overlook other factors affecting service punctuality, such as deviations between actual and scheduled flight departure and arrival times, and ground service delays due to vehicle breakdowns. These uncertainties that arise during airport operations can significantly impact the feasibility and reliability of scheduling plans. Therefore, in the following section, we will delve into the current research on methods for handling uncertainties and their practical applications in scheduling airport specialized vehicles.

### Uncertainty handling methods

In the realm of scheduling specialized vehicles at airports, addressing uncertainties presents a significant challenge. Zhu tackled the uncertainty stemming from fluctuations in flight schedules by employing a normal distribution to statistically measure the time difference between actual and scheduled arrival times. They utilized a chance-constrained approach to describe the uncertainty of actual flight arrival times and integrated the collaborative scheduling of fuel trucks and ferry vehicles, developing a multi-objective mixed-integer programming model^19^. Meanwhile, Han considered the uncertainty of actual flight arrival and departure times and introduced a more realistic distribution of deviations between actual and scheduled times. They proposed a multi-objective two-stage optimization model and constructed a time-space network based on network flow for solving^20^. Additionally, Xu devised a ferry service diversion method considering differences in flight arrival times. Initially, the model was transformed into a dynamic programming stochastic model with chance constraints. Then, the sample average approximation technique was applied to convert the stochastic model into a confident deterministic one^21^. Analyzing the distribution of delay times and arrival times in the flight schedule can enhance the robustness of scheduling plans and optimize ground service for flights. However, statistical distribution patterns might not consider extreme delay situations and could fail to completely capture fluctuations throughout the entire process. Therefore, relying solely on distribution statistics and parameter modeling methods may be overly idealized.

In response to the uncertainty during flight operations, many scholars employ simulation modeling or simulation-optimization methods for handling uncertainty. Simulation, as a means of simulating real-world systems, processes, or events, aids in studying, understanding, and predicting their behaviors and performances. Norin developed a detailed simulation model of the turnaround process, integrating optimization and simulation. Using de-icing vehicles as an example, it was demonstrated that this approach can improve airport logistics efficiency^22^. Fei Proposed a two-dimensional genetic algorithm to address the integrated scheduling of vehicles and personnel. The approach was validated using a Simio simulation model, leading to optimized operational scheduling at the airport^23^. Tomasella Utilized Simheuristic with an agent-based/discrete-event hybrid simulation model to address tactical resource allocation in aircraft turnaround^24^. Guimarans Combined Monte Carlo simulation with metaheuristic optimization algorithm, evaluate the scheduling plans from the simulation results, and guide the algorithm to generate more robust solutions^5^. Gök Considered multiple scheduling activities in airport turnaround, embedded simulation into optimization algorithm, and utilized efficient search-evolution mechanisms to generate more robust solutions^25^. Liu developed a conflict resolution based sim-opt framework, introduced dynamic window to divide the flight zone into multiple sub-intervals , and proposed a matching algorithm based on slack and tight constraints to optimize the possible resource requirements of each vehicle, thereby improving the initial schedule solution’s search depth and reliability^26^.

However, the parameters in these methods are derived from direct assumptions and probabilistic statistical methods. Predefined parameters cannot describe the entire scheduling process and are unable to handle extreme delay scenarios. Additionally, due to the lack of real-time interaction with the actual airport, these methods cannot monitor abnormal events during airport operations or respond rapidly to random disturbances causing flight delays. To address the need for timely response and accurate monitoring of random disturbances^27^, DT technology has been applied to scheduling processes. Compared to traditional scheduling modes, DT-based scheduling achieves a deep integration of physical and virtual spaces, enhancing real-time responsiveness, robustness, and accuracy. Therefore, in the next section, we will discuss the current research status and application areas of DT.

### Digital twins technology

DT has experienced rapid development in recent years. With its virtual-real mapping and real-time interaction, it enables real-time monitoring and quick response to operational uncertainties. It finds widespread application in addressing scheduling problems in factory workshops and production facilities. Zhang proposed a proactive job-shop scheduling strategy driven by DT data to address the information asymmetry phenomenon caused by the latency of manufacturing data transmissions and stochastic events on the physical production site^28^. Fang explored the application of DT in optimizing workshop scheduling and proposed a framework that integrates DT technology with a scheduling algorithm, with a specific focus on optimizing uncertainty in the intelligent manufacturing process^29^. Negri developed a framework that integrates DT to optimize production scheduling in the presence of uncertainty. This framework combines a genetic algorithm and discrete event simulation, providing an efficient solution for scheduling optimization in complex production environments^30^. Li proposed a framework for anomaly detection and dynamic scheduling using DT, optimizing and adjusting scheduling plans in real time through a sliding window mechanism. Combined with an improved grey wolf optimization algorithm, it addresses the problem of workshop scheduling^31^. Ghaleb utilized DT to optimize real-time scheduling processes, effectively addressing real-time changes in production by employing an improved simulated annealing algorithm combined with a mixed-integer programming model^32^.

The initial applications of DT in the aviation industry emerged in aircraft design, prediction, and health maintenance^33^. With the rapid development of related technologies, research on DT in airport operations and safety management has also been increasing. Souanef conducted an analysis of DT in Unmanned Aircraft System (UAS) Traffic Management systems and its impact on future airspace integration, highlighting its crucial role in ensuring the safe, efficient operation, and airspace sharing of unmanned aircraft^34^. Conde proposed a reference concept and data model for airport DT based on standardized interfaces. This model is designed for monitoring and managing aircraft turnaround events, with the aim of enhancing the efficiency of flight turnaround operations^8^. Saifutdinov introduced an ongoing interactive model between DT and airport ground traffic systems. DT serves as a universal information repository, contributing to the development and testing of scheduling solutions in the flight ground-handling domain^35^. Zhang proposed a specialized vehicle real-time scheduling strategy for digital twins built on a cloud-edge computing architecture. It utilizes real-time flight arrival information and current vehicle status to generate and adjust schedules for specialized vehicles^36^.

Based on the above discussion, while there have been certain advancements in the dynamic scheduling and uncertainty handling of specialized vehicles, they lack effective interaction with the actual airport operational environment. This leads to scheduling plans that fail to meet real-world operational demands and are inefficient in handling unexpected events, thereby impacting service quality and operational efficiency. Therefore, we propose to integrate optimization, simulation, and DT to establish a validation and feedback loop between optimization and simulation, which will enhance the reliability of scheduling plans. Additionally, we will leverage DT to provide data support and anomaly detection capabilities, thereby improving the dynamic adjustment capability of scheduling plans during operational processes. Finally, this paper proposes a multi-strategy scheduling framework for specialized vehicles based on digital twins.

## Model construction

### Problem description

All service tasks for specialized vehicles are assigned based on the flight schedule and the ground service requirements. It is necessary to consider the following features comprehensively: whether the flight is arrival or departure *x*, whether it is at a nearby or remote gate *y*, the type of aircraft *T*, the type of specialized vehicle *M*, the capacity of the specialized vehicle $c_{m}^{max}$ , and the service time of the specialized vehicle $ST_{m,t}$. The complete flight ground-handling process includes passenger embarkation and disembarkation, catering, cabin cleaning, water replenishment, wastewater disposal, refueling, cargo loading and unloading, and aircraft push back. These ground service involve the cooperative operations of ferry vehicle, cater truck, clean truck, cleanwater truck, sewage truck, fuel truck, baggage truck, and tractor.

This problem can be formulated as a vehicle routing problem with time windows, with the objective of minimizing the total travel distance and waiting time for vehicles. The service requirements for each flight *i* are different, and the task assignment is based on the demand for vehicle types $l_i$ and the resource requirements for vehicles $a_{i,m}$ . The initial departure location for all specialized vehicles is the depot *P*, and all parking locations are known. After completing all tasks, the vehicles need to return to depot. The arrival time of specialized vehicles for each task $TA_{i,m,j}^{x,y}$ must adhere to the arrival time window [$EAT_{i,m}$,$LAT_{i,m}$] . considering the distance from the depot to the gate $D_{0,i}$, the distance from one gate to another $D_{i,k}$, and the travel speed $v_m$. This enables the calculation of the departure time for each task of the vehicle. Once the preceding service $PreT_{i,m}$ for the specialized vehicle is completed, the ground service for this task can commence. The waiting time $TW_{i,m,j}^{x,y}$ for the vehicle at the aircraft position can be calculated. When the service duration meets the specified average service time $ST_{m,t}$ , it is assessed whether the current resources $c_{m,j}^{cur}$ of the vehicle exceed the resource requirements $a_{i,m}$ for the next task. If not, the vehicle returns to the depot to replenish resources $c_{m}^{max}$ and undergoes rest according to the specified time $RecT_m$.

### Model assumptions

To abstract the complexities of a real-world system and establish a multi-type specialized vehicles scheduling model, several assumptions must be made. These assumptions help reduce the computational complexity of the optimization algorithm, enabling it to find solutions in a shorter time. Without these assumptions, the system may be too complex to compute an optimal solution. The handling of uncertainties will be addressed by the subsequent simulation model.

- All specialized vehicles are centrally managed in the depot, but each vehicle has a different parking location.
- Each service for every flight is completed by a vehicle, and all vehicles returning to the depot need to replenish supplies.
- For aircraft of the same type, the service time for a specific service is the same. This study involves three types of aircraft: small (S), medium (M), and large (L).
- The service time completed by the tractor determines the completion time of all services for departure flights.
- Ferry vehicle, baggage truck, and fuel truck need to return to warehouse to replenish resources after each task, while other vehicles can continuously perform services when resources are sufficient.
- All specialized vehicles are in an available state and free of malfunctions.
- All flights have the same service priority, and the schedule of service tasks is based on the flight schedule.

### Parameters and constraints

#### Parameters description

The model-related parameters are defined in Table 1.

**Table 1:** Parameter and description.

Parameter	Description
*i*, *k*	index of flight, i, k = 1,2,...,n
*j*	index of vehicle, j = 1,2,...,$q_{m}$
*m*, *t*	vehicle type and aircraft type ,$m\in M$,$t\in T$
*M*	the set of specialized vehicle types {ct, gt, wt, bt, ft, st, tt, dt} corresponds to clean truck, fuel truck, cleanwater truck, baggage truck, cater truck, ferry truck, tractor, and sewage truck, respectively
*T*	the set of aircraft types (S, M, L)
*F*	the set of departure and arrival flights
*n*	the number of departure and arrival flights
$q_m$	the number of m type specialized vehicle
*h*	the total number of specialized vehicles at the airport
*x*	when the flight is arrival, *x* is equal to ’A’, otherwise ’D’
*y*	when the gate is a remote gate, *y* is equal to ’R’, otherwise ’C’
$l_i$	the required number of specialized vehicles for flight i
*P*	all specialized vehicle depot
$v_m$	the drive speed of m type specialized vehicle
$c_{m}^{max}$	the maximum capacity of m type specialized vehicle
$c_{m,j}^{cur}$	the current available resources of m type specialized vehicle j
$a_{i,m}$	the resource requirement of flight i for m type specialized vehicle
$FT_{i}^A$	the planned arrival time for flight i
$FT_{k}^{D}$	the planned departure time for flight k
$FST_i^{x,y}$	the allowed start service time for flight i
$ST_{m,t}$	the average service time of m type specialized vehicle to aircraft type t
$TA_{i,m,j}^{x,y}$	the actual arrival time at the gate for m type specialized vehicle j
$TW_{i,m,j}^{x,y}$	the waiting time at the gate for m type specialized vehicle j
$PreT_{i,m}$	the completion time of the pre-service for flight i with m type specialized vehicle
$RecT_m$	the break time of m type specialized vehicle
$EAT_{i,m}$	the earliest allowed arrival time at the gate
*LAT* *i*, *m*	the latest allowed arrival time at the gate
$D_{i,k}$	the distance between flight i and flight k
$D_{0,i}$	the distance between depot and flight i
$D_{i,0}$	the distance between flight i and depot
$u_{i,m,j}$	equals 1 if flight i is served by m type specialized vehicle j, otherwise 0
$d_{i,k,m,j}$	equals 1 if m type specialized vehicle j continues to serve flight k after completing service for flight i, otherwise 0
$e_{i,m,j}$	equals 1 if m type specialized vehicle j returns to the depot after servicing flight i, otherwise 0
$q_{i,m,j}$	equals 1 if m type specialized vehicle j departs from the depot to service flight i,otherwise 0
$z_{i,m}$	equals 1 if flight i requires service from m type specialized vehicle, otherwise 0

#### Objective function and constraints

In this paper, we divide the objective function of the problem into two parts. Firstly, the first objective is to minimize the total vehicle travel distance. This objective not only reduces operational costs from an economic perspective but also helps decrease the travel time of vehicles, effectively shortening the overall airport delay time. It is described by formula (1). Secondly, the second objective is to minimize the waiting time of vehicles at the gate. If vehicles arrive at the gate too early, it may result in a decrease in the efficiency of both vehicles and manpower resources. By effectively reducing waiting time, we can not only improve the utilization of vehicles during peak periods but also achieve a more compact and efficient vehicle scheduling plan with a limited number of vehicles. Formula (2) is employed to describe this objective,$w_1$ and $w_2$ represent the different weights for the waiting times of departure and arrival flights, respectively . We particularly consider the modeling differences between the optimization model and the simulation program, which may lead to a certain degree of delay in the scheduling plan. However, these delays are generally within an acceptable range. The model design pays special attention to flight delay times, treating it as a key indicator for evaluating the feasibility of the scheduling plan. However, minimizing delay times is not treated as an independent optimization objective but rather as a fundamental condition for the feasibility of the scheduling plan. This approach balances the practicality and efficiency of the model, ensuring its reliability and effectiveness in real-world environments.

1 $$\begin{aligned}{}&f(D)=\min \sum _{i=1}^n\sum _{m=1}^Mz_{i,m}({e}_{i,m,j}* D_{0,i}+{q}_{i,m,j}* D_{i,0}+d_{i,k,m,j}* D_{i,k}) \end{aligned}$$

2 $$\begin{aligned}{}&\quad f(T) = \min \sum _{i=1}^n\sum _{m=1}^M w_1* TW_{i,m,j}^{A,y}+w_2* TW_{i,m,j}^{D,y} \end{aligned}$$

3 $$\begin{aligned}{}&\quad \sum _{j=1}^{q_m} \mu _{i,m,j} = z_{i,m}, \quad i\in F, m\in M \end{aligned}$$

4 $$\begin{aligned}{}&\quad \sum _{m=1}^{M} z_{i,m} \sum _{j=1}^{q_m} \mu _{i,m,j} = l_i, \quad i\in F \end{aligned}$$

5 $$\begin{aligned}{}&\quad \sum \limits _{i=1}^{n}l_i=\sum \limits _{i=1}^{n}\sum \limits _{m=1}^{M}\sum \limits _{j=1}^{q_m}\mu _{i,j,m} \end{aligned}$$

6 $$\begin{aligned}{}&\quad c_{m}^{max}=1, m\in \{bt,st,ft\}\subset M \end{aligned}$$

7 $$\begin{aligned}{}&\quad e_{i,m,j}=0,i\in F,m\in\{bt,st,ft\}\subset M \end{aligned}$$

Constraints (3)-(7) represent the vehicle service and fleet constraints. Constraint (3) ensures that at any given moment, a specific type of service is provided by only one specialized vehicle, avoiding service overlap. Constraint (4) restricts the number of vehicles providing services for each flight to not exceed a set upper limit $l_{i}$, optimizing resource utilization. Constraint (5) ensures that the total amount of services received by all flights equals the total usage of vehicles, guaranteeing comprehensive coverage of all service requirements. Constraints (6) and (7) specify that ferry vehicle, baggage truck, and fuel truck need to return to parking spaces to replenish resources after each task, while other vehicles can provide continuous service until resources are exhausted.

8 $$\begin{aligned}{}&\sum_{i=1}^nd_{0,i,m,j}=\sum_{i=1}^nu_{i,m,j},m\in M,j\in q_m \end{aligned}$$

9 $$\begin{aligned}{}&\quad \sum_{i=1}^nd_{0,i,m,j}=\sum_{k=1}^n(1-d_{k,0,m,j}),m\in M,j\in q_m \end{aligned}$$

Constraints (8) and (9) pertain to depot constraints. Constraint (8) states that each vehicle must have a clearly assigned task before departure to ensure the effective utilization of resources. Constraint (9) specifies that vehicles will return to depot when their capacity is depleted or when all tasks are completed, preventing them from lingering at aircraft gates or elsewhere.

10 $$\begin{aligned}{}&\max _{m\in \{ct,gt,ft\}\subseteq M}\left\{ TA_{i,m,j_1}^{Dy}+TW_{i,m,j_1}+ST_{m,t}\right\} \le TA_{i,st,j_2}^{Dy}+TW_{i,st,j_2}, i\in F, j_1\in q_{m}, j_2\in q_{st} \end{aligned}$$

11 $$\begin{aligned}{}&\quad TA_{i,st,j_1}^{A,R}+TW_{i,st,j_1}+ST_{s,t} \le TA_{i,ct,j_2}^{A,R}+TW_{i,ct,j_2}, i\in F, j_1\in q_{st}, j_2\in q_{ct} \end{aligned}$$

12 $$\begin{aligned}{}&\quad \max _{m\in M/tt}\left\{ TA_{i,m,j_1}^{\textrm{x,y}}+TW_{i,m,j_1}+ST_{m,t}\right\} \le TA_{i,tt,j_2}^{\textrm{x,y}}+TW_{i,tt,j_2}, i\in F, j_1\in q_m, j_2\in q_{tt} \end{aligned}$$

13 $$\begin{aligned}{}&\quad EAT_{i,m} \le TA_{i,m,j}^{\textrm{x,y}} \le LAT_{i,m}, i\in F,m\in M, j\in q_m \end{aligned}$$

14 $$\begin{aligned}{}&\quad TA_{i,m,j}^{x,y}+TW_{i,m,j}^{x,y}+ST_{m,t}+D_{i,0,m,j}/\nu _{m}+RecT_{m} \le TA_{k,m,j}^{x,y}-D_{0,k,m.j}/\nu _{m}, i,k\in F,m\in M,j\in q_{m} \end{aligned}$$

15 $$\begin{aligned}{}&\quad \min \{TA_{i,m,j}^{x,y}+TW_{i,m,j}^{x,y}\} \le FST_i, i\in F, m\in M, j\in q_m \end{aligned}$$

16 $$\begin{aligned}{}&\quad TA_{i,m,j}^{A,y}+TW_{i,m,j}^{A,y} = \max \left\{ TA_{i,m,j}^{A,y},FT_{i}^{A},PreT_{i,m}\right\} , i\in F, m\in M, j\in q_{m} \end{aligned}$$

17 $$\begin{aligned}{}&\quad TA_{i,m,j}^{D,y}+TW_{i,m,j}^{D,y} = \max \left\{ TA_{i,m,j}^{D,y},PreT_{i,m}\right\} , i\in F, m\in M, j\in q_m \end{aligned}$$

Constraints (10)-(17) are related to time window constraints. Constraint (10) indicates that the ferry vehicle for departure flights from remote gates can only start its service after vehicles like cater truck, clean truck, and fuel truck have completed their services. Constraint (11) states that the clean truck for arrival flights at remote gates can only start its service after the ferry vehicle has completed its service. Constraint (12) specifies that for departure flights, the tractor must start its service only after other vehicles have completed their services. Constraint (13) ensures that the arrival time of any vehicle at the parking position falls within the allowed time window. Constraint (14) states that if a vehicle undertakes multiple service tasks, its next departure time should be later than the time it completed the previous service or its rest time. Constraint (15) indicates that the start time of all service vehicles for a flight should be later than the allowed service start time for the flight. Constraint (16) states that the start time of service vehicles for arrival flights is determined by the maximum among three values: the vehicle’s arrival time at the parking position, the planned arrival time of the flight, and the time when the preceding service vehicle completes its service. The preceding service refers to the service that must be completed before the vehicle in question starts its service. For example, for arrival flights at remote gates, the clean truck must start after the refueling vehicle completes its service. Constraint (17) specifies that the start time of specialized vehicles for departure flights depends on the later of the vehicle’s arrival time at the parking position and the time when the preceding service is completed.

18 $$\begin{aligned}{}&q_{i,m,j}*c_{m,j}^{cur}=q_{i,m,j}*c_{m}^{max},i,k\in F,m\in M,j\in q_m \end{aligned}$$

19 $$\begin{aligned}{}&\quad z_{i,m}c_{m,j}^{cur} \ge z_{i,m}a_{i,m},i\in F,m\in M,j\in q_m \end{aligned}$$

20 $$\begin{aligned}{}&\quad \textbf{e}_{i,m,j},d_{i,k,m,j},q_{i,m,j},z_{i,m}\in \{0,1\},i,k\in F,m\in M,j\in q_m \end{aligned}$$

Constraints (18)-(19) are resource capacity constraints. Constraint (18) specifies that if a vehicle returns to the depot, it must replenish its supplies. Constraint (19) states that the vehicle’s existing resources must be greater than or equal to the resource requirements of the service flight; otherwise, the vehicle cannot provide service. Constraint (20) specifies that the decision variable values can only be 0 or 1.

## Multi-strategy scheduling framework for specialized vehicles based on digital twins

**Figure 2:**
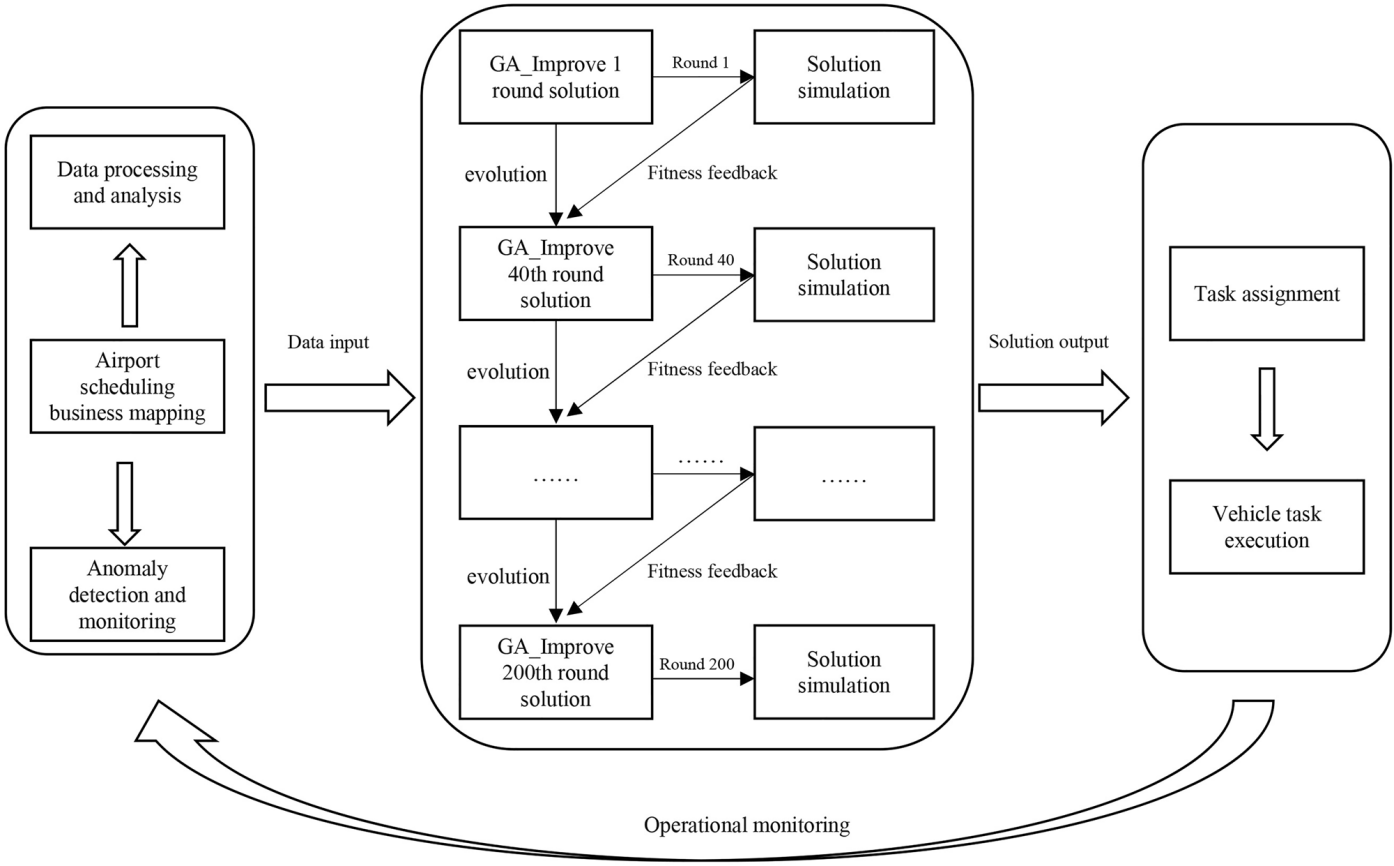
Main steps of a digital twins-based multi-type vehicles collaborative scheduling.

In this paper, we propose a digital twin-based multi-strategy framework to optimize the collaborative scheduling of specialized vehicles in airport. With the support of DT technology, real-time monitoring and situational awareness of the airport are achieved, enabling the acquisition of operational status and equipment information. By analyzing both real-time and historical data, this framework assists decision-makers in making more informed scheduling decisions. The implementation of this framework includes the following steps, as illustrated in Fig. 2. Firstly, a virtual model of the flight ground-handling operations is established based on the digital twins. This virtual model continuously monitors the operational status of the airport and is constantly updated to remain consistent with the physical airport. The virtual model manages and updates data, including real-time and historical data, such as flight information, the health status of specialized vehicles, and personnel information. Subsequently, these data are cleaned, processed, and sent to the optimization module. An improved genetic algorithm is employed to iteratively solve the scheduling problem, with 200 iterations round for the evolution of the algorithm. During the evolution process, five rounds of simulation validation are conducted to provide fitness feedback to the algorithm, guiding its evolution. For the iterations without simulation validation, the objective function in the model serves as the fitness indicator to guide the algorithm’s evolution. Finally, the optimal solution is output to the scheduling center, where task assignments are made according to the scheduling plan. Vehicles begin servicing flights based on the assigned tasks, and the digital twin continuously updates the virtual model to synchronize the virtual and physical spaces. When new scheduling demands arise or significant deviations occur in the current scheduling plan, a new scheduling plan is generated based on real-time data, achieving a closed-loop iteration between the virtual and physical spaces.

**Figure 3:**
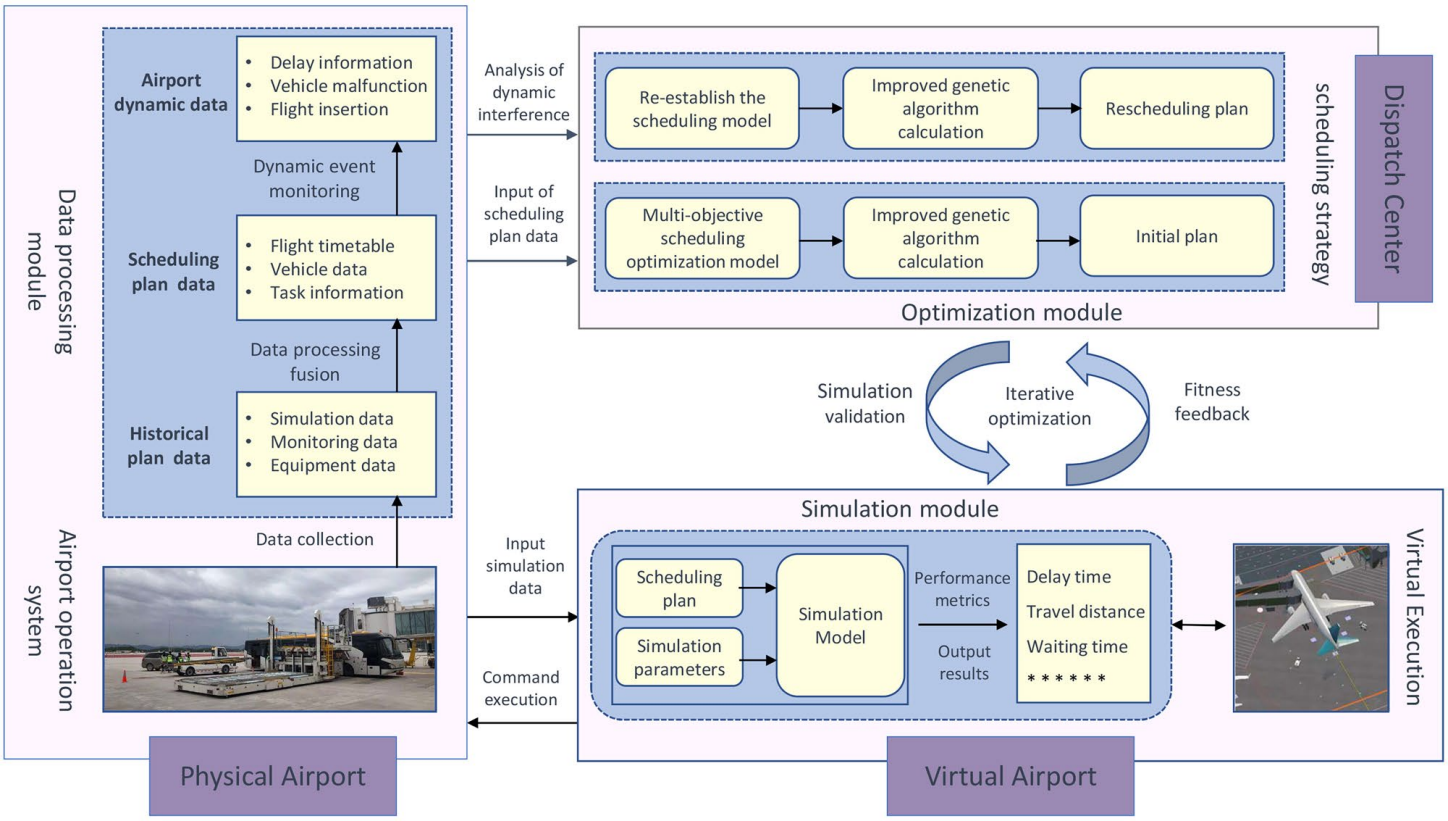
Multi-strategy scheduling framework for specialized vehicles based on digital twins.

The framework consists of three components: physical space, virtual space, and the schedule center, as illustrated in Fig. 3 . The communication of operational data, generated by corresponding processing modules within the framework, facilitates two-way interaction and iterative updates among the modules. This process results in the generation of more optimized and robust scheduling solutions. Specifically, the data processing module in the physical space is responsible for handling and modeling historical data, scheduling input data, and real-time airport dynamic data. It provides necessary scheduling data and simulation parameters to both the optimization module in the scheduling center and the simulation module in the virtual space. Subsequently, based on scheduling requirements, the optimization module executes corresponding scheduling strategies to generate a solution, which is then input into the simulation module for near real-time verification. Additionally, the scheduling plan can undergo process demonstrations in the virtual space, with the simulation results serving as evaluation metrics to guide the optimization algorithm toward the optimal solution. The continuous interaction and iteration between the optimization module and the simulation module eventually form an executable scheduling plan. Finally, the scheduling plan is output to the physical space for actual execution. The data processing module continuously updates airport operational data, calculating the deviation between real-world operations and those in the virtual space to dynamically adjust the scheduling plan. In the event of significant delays, the framework can utilize real-time data to generate a rescheduling plan to cope with unforeseen circumstances.

DT is the core component enabling the closed-loop operation of the scheduling framework, integrating the physical space, virtual space, and the scheduling center. During the framework’s operation, it also integrates the optimization algorithm and the simulation model. Traditional simulation-optimization frameworks typically involve a one-way feedback loop from optimization to simulation, merely validating the performance of solutions in different scenarios. In contrast, our approach integrates DT, optimization algorithms, and simulation models, providing a two-way feedback mechanism between the optimization algorithm and the simulation model, supported by data from the DT. The DT supplies the necessary data support, while the optimization algorithm generates solutions and the simulation model validates these solutions, returning fitness values to guide and enhance the algorithm’s exploratory capabilities. Concurrently, the DT analyzes data to provide more accurate simulation parameters. This interaction between the physical and virtual spaces facilitates self-optimization and self-adaptation within the entire framework. DT provides precise data and parameters to the optimization algorithm and simulation model. The data generated by these components is stored in the virtual model, offering valuable references for future framework operations. Within the virtual model, the DT monitors the deviations between the virtual execution of the scheduling plan and the actual airport operations, visually presenting the simulation results and real operational states to airport staff. Moreover, the DT can detect anomalies, such as flight delays, equipment failures, or vehicle resource shortages. Upon identifying these anomalies, the system issues proactive alerts to the staff and provides real-time data analysis. Leveraging the optimization algorithm and the simulation model, a rescheduling plan is promptly generated to address these issues.

### Data processing module

In the initial stage of the specialized vehicle scheduling system, the focus is on providing initial computational data to support the improved genetic algorithm calculations in the optimization module. Firstly, utilizing the flight schedule timetable as the basis for generating the specialized vehicle service plan, the planned time is considered as the actual departure or arrival time of the flight to ensure the accuracy of the initial plan. Secondly, the vehicle resource table provides critical information such as the available quantity of vehicles, resource-carrying capacity, and service time. These data are rigorously considered when matching service flights. Additionally, the location information table contains detailed distance data between parking positions and between parking positions and gates. Based on the actual airport layout, this table avoids the use of simplified models, thereby enhancing the feasibility of the scheduling plan.

During operation, real-time data processing becomes crucial due to potential parameter updates and dynamic disturbances. The updated parameters output by the simulation module, serving as evaluation metrics for the optimization algorithm, need to undergo precise handling when input into the optimization module. Furthermore, in the face of disruptions such as flight delays, vehicle malfunctions, and flight changes, the system must promptly detect and address this information to trigger rescheduling strategies and generate a new scheduling plan.

### Simulation module

**Figure 4:**
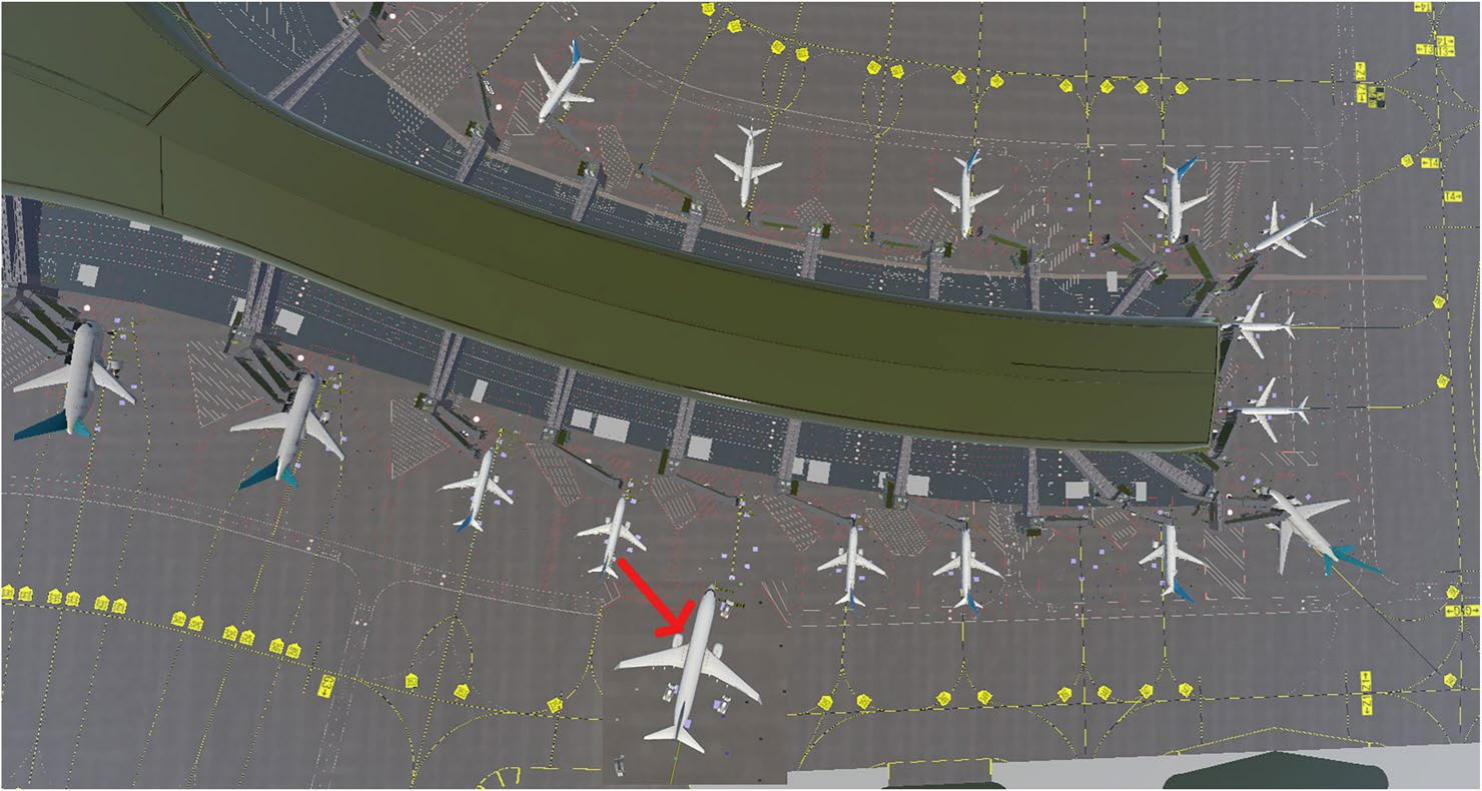
Smulation scenario.

In this paper, we generate random or realistic airport operational scenarios by adjusting simulation parameters in Unity(Unity 2021.3.23f1,https://unity.com/cn). Considering that scheduling solutions generated by the genetic algorithm perform well in idealized airport environments but may fail in real airport environments due to random disturbances such as flight delays and specialized vehicle malfunctions, we seek to enhance the robustness of the solutions. Although some disturbance parameters are incorporated into the optimization algorithm, the robustness of the solutions is still insufficient without comprehensive simulation program validation. Therefore, we create corresponding operational scenarios based on actual airport operations in the Unity(Unity 2021.3.23f1,https://unity.com/cn) and implement an efficient simulation program using C#,as show in Fig. 4.

Initialization and computation of simulation scenes require the use of simulation parameters and schedule plans. The initialization setting of the simulation scene is crucial to ensure a high degree of similarity between the simulation environment and the real environment. For instance, the initial positions of specialized vehicles should be distributed reasonably, and the pathfinding algorithm for vehicles should be efficient. The scheduling plan provides information about the service flights for specialized vehicles and the order of service. Therefore, it is essential to use the vehicle service time specifications and flight schedule table from the simulation program to calculate the initial start and completion times for vehicle operations. Due to the continuity of ground services at the airport, delays in vehicle operations can easily lead to chain reactions,thus, the program needs to continuously update the time parameters of vehicles during runtime to avoid conflicts and resource wastage until the operations are completed.

In the simulation program, we particularly address the problem of delay propagation caused by the extension of vehicle service times beyond the specified time. When service time of a vehicle is prolonged, it may cause delays in other services for the same flight and subsequent services for the vehicle. Delay propagation in this context can be categorized into two types: first, for different services within the same flight, there are rules for parallel and sequential execution. The delay of a vehicle providing sequential services will affect the timeliness of subsequent services. Second, when a vehicle serves multiple flights, if the completion time of the current service exceeds the start time of the next service, it leads to operational conflicts for the same vehicle, affecting the subsequent flights. To address these delays, our simulation program employs the application of a shifted service start time and a buffer window. For the first type, the affected vehicle’s service time is adjusted based on the completion time of the preceding service, no longer limited to the originally scheduled time window, and any violations of time windows are recorded. For the second type, the vehicle’s service start time is updated to the completion time of the previous service, and updates are made to the time parameters of other affected vehicle services, with relevant violations recorded. Although delay propagation can have widespread effects, the presence of time buffers between service time windows and different services allows us to absorb some delays, thereby positively contributing to the adjustment of flight services. As shown in Algorithm 1.

Finally, we validate the robustness of the scheduling plan by adjusting disturbance parameters in the simulation program and conducting multiple parallel computations. During this process, we focus on the number of time window violations and the corresponding delay times in the simulation, i.e., the difference between the actual service completion time and the latest allowed completion time. Additionally, we calculate the total travel distance and total waiting time of vehicles in the scheduling plan, where the latter represents the time difference between the vehicle arrival at the designated location and starting the service. Through these comprehensive simulation data, we can thoroughly assess the robustness of the scheduling plan. Furthermore, these simulation results are fed back into the optimization algorithm as crucial reference points for fitness values, guiding the optimization algorithm to search more efficiently. This iterative process not only ensures the reliability of the scheduling plan but also improves the overall efficiency of the scheduling process.

**Algorithm 1:**
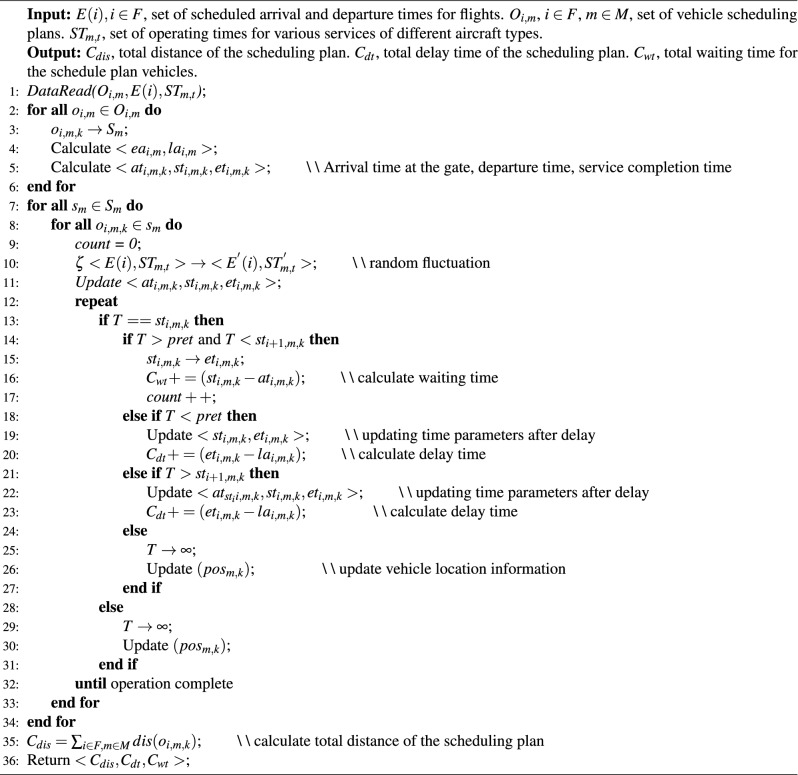
Simulation procedure.

### Optimization module

The scheduling model established in this paper aims to solve the problem of cooperative scheduling for multi-type specialized vehicles. Due to the involvement of various types of vehicles and aircraft, and the existence of complex service rules in terms of timing for both same-type and different-type vehicles, it becomes challenging to represent the solution in a simple and reasonable encoding format. Genetic algorithms, as optimization algorithms based on the principles of biological evolution, have been widely used to solve complex optimization problems. Their strong search capability and adaptability make them perform well when faced with multi-constraint and multi-objective problems. However, traditional single-layer or double-layer encoded genetic algorithms are more suitable for single-type vehicle scheduling problems. When dealing with highly complex multi-type vehicles cooperative scheduling problems, they may face challenges in efficiency and solution quality. Using single-layer or double-layer encoding would complicate the expression of solutions, where each encoded value might represent more information. This complexity can lead to infeasible solutions or local optima during the crossover and mutation operations of the genetic algorithm, reducing the algorithm’s exploration ability and the stability of population evolution. To address these encoding issues, we designed an encoding scheme that can comprehensively describe vehicle numbers, service sequences, and service stages. We proposed an improved genetic algorithm based on a three-layer hybrid encoding structure to solve the scheduling model. In the crossover operation of the genetic algorithm, we introduced a local evaluation factor, and in the mutation operation, we designed a collaborative mutation mechanism to maintain stability and diversity in the population evolution process. The proposed improved algorithm is better suited for exploring optimal solutions in the complex space of multi-objective optimization problems.

#### Chromosome encoding and decoding

**Figure 5:**
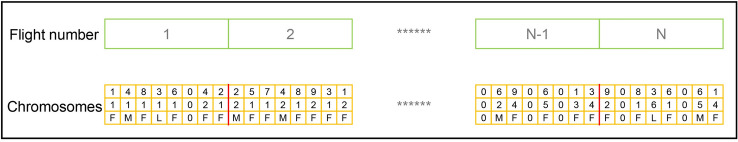
Three-layer chromosome structure.

We proposes an innovative three-layer chromosome structure to represent service information in airport vehicle scheduling. The unique structure includes flight numbers, service sequences, and service stages. To express this information more intuitively and effectively, a hybrid encoding approach using natural number encoding and symbol encoding is employed,this encoding method is more concise and undergateable compared to traditional methods. As shown in Fig. 5, each chromosome is encoded as a tuple of length 24N, divided into three layers. The first layer (1 to 8N) is used to specify the allocation of vehicles, the second layer (8N+1 to 16N) represents the service sequence of vehicles, and the third layer (16N+1 to 24N) defines the service stages of vehicles. Different stages are represented by different symbols; for example, ’F’ indicates a vehicle departure from the depot to the gate, ’M’ represents moving from one gate to another, and ’L’ signifies returning from the gate to the depot. It is worth noting that the digit ’0’ in the chromosome indicates that the corresponding flight does not require a specific ground service. For example, if a segment of the chromosome for a flight indicates it is a departure near-gate flight, it might only require services other than ferry service. Specifically, a chromosome substring such as (1,4,8,3,6,0,4,2) denotes the vehicle numbers assigned to various services, and (2,1,1,3,1,0,2,1) indicates the service sequence of these vehicles throughout the scheduling plan, determined based on the flight service time. Finally, the substring (F,M,F,L,F,0,F,F) with symbols describes the service stages, starting positions and destinations of the vehicles.

#### Population initialization

Initialize settings such as the population size, crossover rate, mutation rate, and number of iterations. Based on the population size, generate the corresponding number of chromosomes, and calculate their respective fitness values to assess the quality of the chromosomes. Sort all chromosomes based on fitness for further operations.

#### Selection operation

In this paper, the top 20 chromosomes in each iteration are preserved and excluded from crossover and mutation operations. The purpose is to ensure that high-quality chromosomes are not disrupted during the evolution process, allowing them to be inherited by the next generation.

#### Improved crossover operation

Crossover operation is a crucial step in genetic algorithm, involving the exchange of genetic material between two chromosomes to generate a new chromosome. Due to the relatively complex chromosome structure in this paper, a random segment crossover method was chosen as the crossover strategy. The aim is to enhance the diversity of offspring chromosomes, thereby improving the exploration ability of the entire algorithm and avoiding local optima.

**Figure 6:**
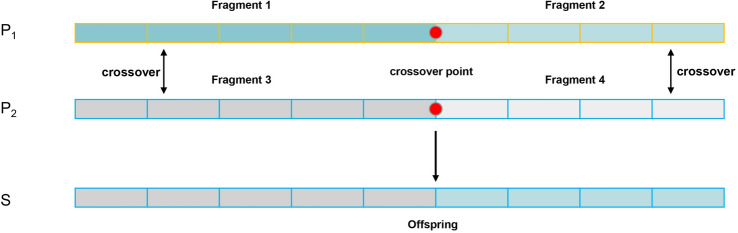
Crossover operation.

**Figure 7:**
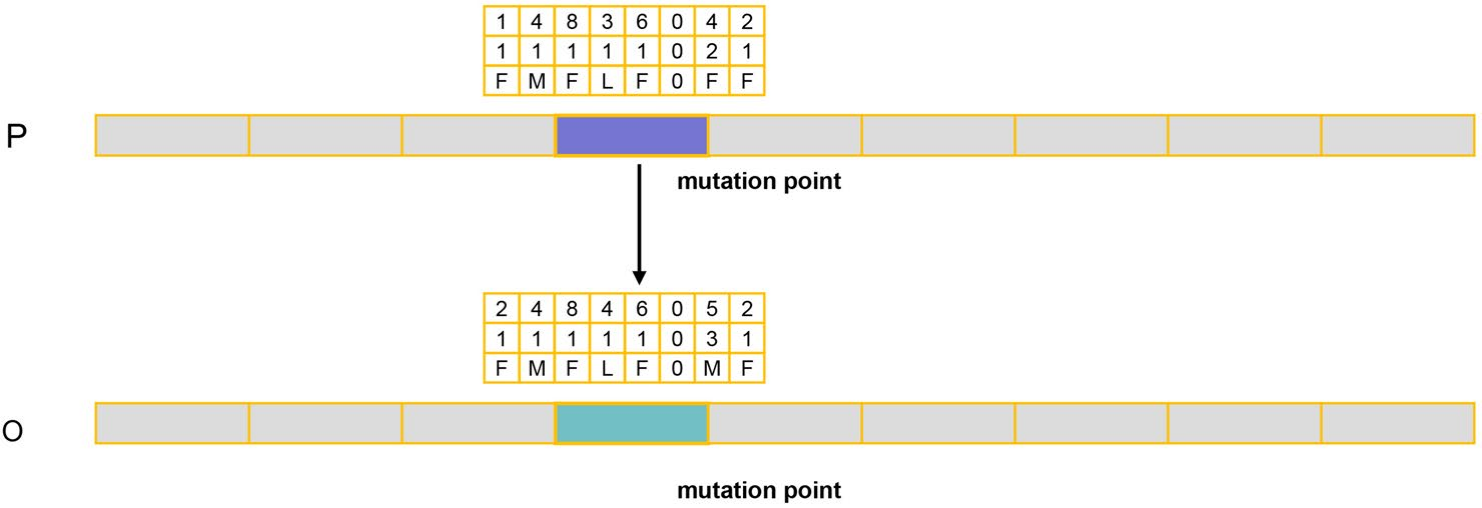
Mutation operation.

Using “Q” to represent the local evaluation factor of chromosome segments, the two chromosomes have a total of four chromosome segments to determine the offspring chromosome after the crossover operation, as shown in Fig. 6 . The local evaluation factor is the weighted average of the chromosome segment’s travel distance and vehicle waiting time. Through the local evaluation factor, it can be determined how the chromosomes should cross to obtain a better solution.

21 $$\begin{aligned} Q_{P_{i,j}}&= w\frac{D(P_{i,f})}{D(P_1)+D(P_2)} + (1-w)\frac{T(P_{i,f})}{T(P_1)+T(P_2)} \end{aligned}$$

The formula (24) for calculating the total travel distance of a chromosome segment, where “m” represents the specialized vehicle type, “j” represents the vehicle number, and “c” and “k” represent the flight numbers.

22 $$\begin{aligned} {\left\{ \begin{array}{ll} \begin{aligned} c &{}= 1, &{} t &{}= \text {pos} - 1, &{} f &{}= 1 \\ c &{}= \text {pos}, &{} t &{}= N, &{} f &{}= 2 \end{aligned} \end{array}\right. } \end{aligned}$$

23 $$\begin{aligned} D(P_{i,f})&= \sum _{i=1}^n\sum _{m=1}^Mz_{i,m}({e}_{i,m,j}* D_{0,i}+{q}_{i,m,j}* D_{i,0}+d_{i,k,m,j}* D_{i,k}) \end{aligned}$$

The simplified formula (25) for calculating the total waiting time of a chromosome segment for vehicles. Here, “m” represents the specialized vehicle type, “j” represents the vehicle number, and “c” and “k” represent the flight numbers. Additionally, “x” represents arrival or departure, and “y” represents near-apron or far-apron.

24 $$\begin{aligned} T(P_{i,f}) = \sum _{c}^{t}\sum _{m=1}^{M}TW_{c,m,j}^{x,y} \end{aligned}$$

#### Improved mutation operation

Mutation is a means to enhance population diversity and exhibits strong exploratory capabilities,as shown in Fig. 7. In order to maintain overall evolutionary stability, many studies opt for a very small mutation rate. However, there exists a trade-off between population stability and diversity, potentially resulting in limited impact of the mutation operation on the later stages of population evolution. Moreover, single-vehicle mutation shows minimal improvement on the three-layer chromosome structure in this paper. To address this, the research introduces a synergistic mutation mechanism that simultaneously considers both population stability and diversity. This approach aims to elevate population diversity while preserving stability, thereby enhancing the exploratory potential of the optimization model in later stages. The following formula is employed to express this concept: the mutated chromosome is retained for the next iteration when E(i) is greater than 0.

25 $$\begin{aligned} E(i)&= \alpha (F(i)-F(i-1)) + (1-\alpha )(V(i)-V(i-1)) \end{aligned}$$

In terms of preserving population stability, the mutation operation also utilizes a local evaluation factor to determine the generation of offspring chromosomes. The evaluation value is calculated only for the mutated flight point. Random mutation operations may generate infeasible solutions, rendering them unable to run smoothly in the simulation program, therefore, it is necessary to consider both vehicle and flight time window constraints during mutation to ensure the generation of feasible solutions. These constraints not only contribute to maintaining population stability but also accelerate the evolutionary speed of the population, preventing it from getting stuck in local optima.

26 $$\begin{aligned} F(i)&= wD(i) + (1-w)T(i) \end{aligned}$$

In terms of preserving population diversity, enhancing population diversity can expand the exploration space of solutions, particularly in the later stages of the algorithm where continuous optimization of solutions is crucial. For this study, population diversity is defined as the balanced utilization of vehicles. Here, $n_{m,j}$ and $n_{m,k}$ represent the usage frequency of ’m’ type vehicle of ’j’,’k’ ,respectively, and $q_{m}$ represents the total number of vehicles of ’m’ type . This definition ensures that more vehicles can participate in the service, contributing to increased diversity in the population.

27 $$\begin{aligned} V(i)&= \sum _{m=1}^{M}\sum _{j=1}^{q_m}\left( n_{m,j} - \frac{\sum _{k=1}^{q_m}n_{m,k}}{q_m}\right) ^{-2} \end{aligned}$$

#### Algorithm process

The specific steps of the algorithm designed to solve the model in this paper are algorithm 2.

**Algorithm 2:**
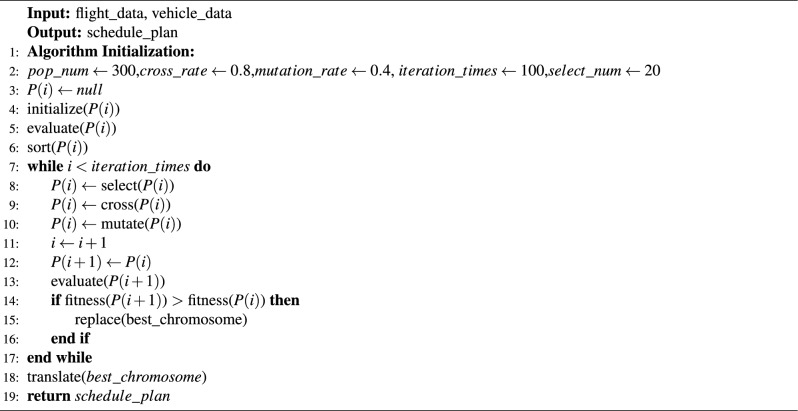
Improved genetic algorithm.

## Case study

To verify the effectiveness and robustness of our proposed Multi-strategy scheduling framework for specialized vehicles based on digital twins, we conducted a case study involving the scheduling of 40 flights and 8 types of specialized vehicles within 3 hour timeframe.

### Processing of experimental data

#### Airport layout

**Figure 8:**
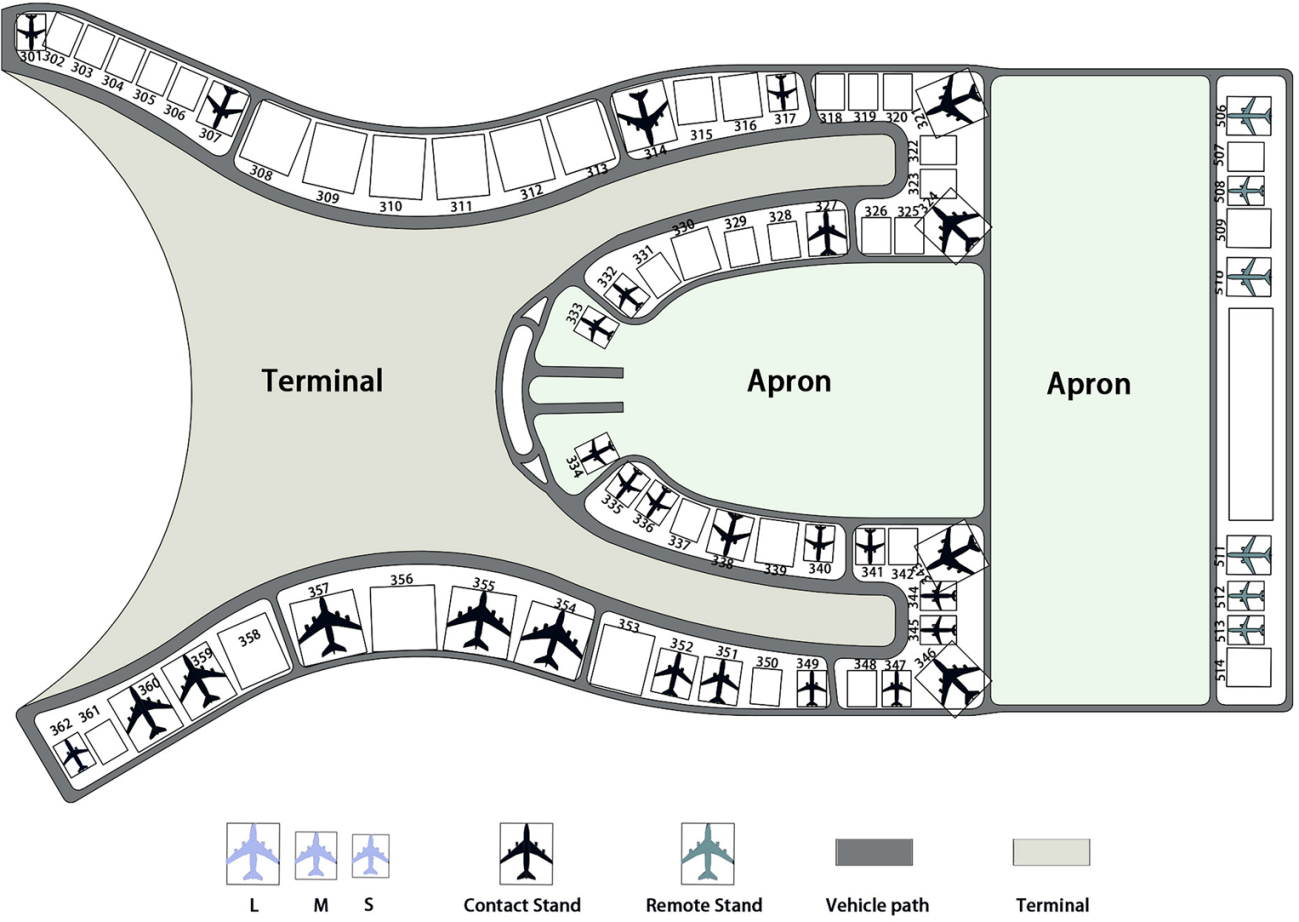
Partial airport gates and routes distribution.

We utilized a specific area of a domestic airport, comprising the terminal building, aircraft parking areas, and taxiways. From a spatial layout perspective, parking spaces are primarily concentrated on one side of the terminal building outline and on the side with jet bridges. The travel distance of the vehicles is not fixed, necessitating the use of pathfinding algorithm to calculate specific distances. Based on the vehicle’s speed, departure location, and arrival location, we can use this distance data to calculate travel time and travel distance. The specific airport layout is illustrated in Fig. 8.

#### Flight detailed data

This study selected the flight data of a domestic airport during the peak period from 6:00 AM to 9:00 AM as the research subject. During this time frame, the airport handled a total of 49 flights, including arrival and departure flights. However, based on the scheduling model and service requirements of this study, we filtered out 40 flights as the research sample, comprising 15 arrival flights and 25 departure flights. The selected flight information is comprehensive, including flight number, aircraft type, planned departure or arrival time, average delay time, flight attribute (arrival or departure), parking gate number, and the attribute of being a near gate or a remote gate. The specific flight information is shown in Table 2.

**Table 2:** Flight data table.

Index	Flight number	Aircraft type	Plan time	Average delay	Departure/Arrival	Park number	Remote/Near
1	8L9863	S	6:00	16 min	A	362	N
2	PN6327	M	6:05	14 min	D	361	N
3	PN6285	L	6:10	21 min	D	511	R
4	PN6314	L	6:15	10 min	A	358	N
5	PN6247	M	6:20	12 min	D	359	N
6	PN6271	M	6:30	11 min	D	514	R
...	...	...	...	...	...	...	...
38	9C8613	S	8:55	10 min	A	355	N
39	CZ5588	S	9:00	6 min	D	356	N
40	HU7343	L	9:00	13 min	A	513	R

#### Vehicle data

In this paper, when considering flight ground-handling process, the focus was primarily on the key specialized vehicles in three main service lines. These vehicles include fuel truck, ferry vehicle, cater truck, cleanwater truck, sewage truck, baggage truck, clean truck, and tractor. The service time for each vehicle varies based on the aircraft type being serviced, with larger aircraft generally requiring longer service times. Additionally, different specialized vehicles have varying capacities for carrying resources, and they can perform single or consecutive services. The specific information about these vehicles, including their types, capacities, and service characteristics, is detailed in Table 3.

**Table 3:** Vehicle attributes and information.

Vehicle type	Service time(S,M,L)(min)	Total number	Service capacity	Drive speed(km/h)
Fuel truck	20, 25, 30	12	1	25
Ferry vehicle	8, 10, 12	6	1	30
Cater truck	20, 25, 30	15	2	35
Cleanwater truck	20, 25, 30	15	3	35
Sewage truck	22, 25, 30	16	2	35
Baggage truck	30, 35, 45	28	1	30
Clean truck	20, 25, 30	20	2	30
Tractor	6, 8, 10	8	3	30

### Experimental setup

#### Parameter configuration

This series of experiments were conducted on a computer with a 64-bit Windows 10 operating system, equipped with a 3.2 GHz CPU and 8 GB RAM. In these experiments, the genetic algorithm was developed and executed using the Python language in the Pycharm integrated development environment. Meanwhile, the simulation program was written in the C# language on the Unity software platform. Considering the numerical interaction requirements between the optimization model and the simulation model, after comprehensive multiple rounds of experimental verification and time efficiency considerations, precise parameter adjustments were made to the improved genetic algorithm. Specifically, the algorithm’s population size was set to 200, the selection size to 20, and the crossover and mutation probabilities were set to 0.8 and 0.4, respectively. In addition, for consistency and comparability of the experiments, the number of iterations for the algorithm was fixed at 100, aligning with the initial population size and iteration number of other comparative algorithm. The simulation program introduced a series of random disturbance factors to more realistically simulate the dynamics and uncertainties of airport operations. These disturbance factors included fluctuations in flight arrival and departure times based on actual delay data, uncertainty in the operation duration of service vehicles, and variability in driving routes. This design aimed to comprehensively assess the adaptability and robustness of the algorithm in dealing with the complexity and uncertainty of actual airport operations.

#### Comparative algorithm

We will compare the experimental results of the proposed GA_Improve with FCFS (First Come First Serve), PSO (Particle Swarm Optimization), ACO (Ant Colony Optimization), and GA (Genetic Algorithm).

FCFS : this is a basic scheduling algorithm that processes tasks in the order of their arrival, without considering the importance or urgency of tasks. It is simple to implement but may not be efficient when dealing with tasks of varying importance or resource requirements. It can lead to resource wastage and longer waiting times.

PSO: this is an optimization algorithm based on swarm intelligence. In PSO, each solution is considered a “particle,” and these particles move in the solution space, searching for the global optimum by tracking individual and swarm’s best-known positions. PSO is particularly suitable for optimization problems in continuous spaces, effectively exploring a large solution space while avoiding local optima.

ACO : this simulates the foraging behavior of ants and is well-suited for solving pathfinding and graph optimization problems. In ACO, ants move on a graph structure, searching for the optimal path from the starting point to the endpoint. ACO excels in finding high-quality solutions in complex search spaces and demonstrates good adaptability to dynamic problems.

GA : this is a search algorithm that simulates natural selection and genetic principles observed in biological evolution. In GA, solutions are treated as individuals, evolving through genetic operations like selection, crossover, and mutation to generate a new generation of solutions. GA’s strengths lie in its diversity and powerful global search capabilities, allowing it to find effective solutions in complex and multimodal search spaces. However, parameter settings significantly influence algorithm performance.

### Experimental analysis

As described above, the digital twins scheduling framework consists of two scheduling strategies at the scheduling center, corresponding to different stages in the scheduling process. It requires comprehensive consideration of the service objects and requirements of specialized vehicles. The data processing module is utilized to gather complex data, including flight schedules, historical flight information, and vehicle usage details. The optimization module and simulation module iteratively optimize to generate robust scheduling plans. The simulation module performs real-time verification of the plans in a virtual space. In case of significant deviations between the virtual space and the actual airport operation, simulation data is output to the data processing module for data integration, providing support for rescheduling at the scheduling center. Therefore, we conducts experiments based on the two scheduling modes of the scheduling center, involving three different tests to validate our method. Firstly, in a deterministic scenario, different algorithms are compared to validate the feasibility of the improved genetic algorithm. In this case, simulation is only used for plan verification and does not participate in the global search of the optimization algorithm. Secondly, in an uncertain environment, the proposed simulation-optimization method is applied to solve the vehicle scheduling problem, aiming to enhance the robustness of the solutions. Finally, in a scenario where a sudden event causes a massive delay, the digital twins-based simulation-optimization method generates rescheduling plans within the digital twins system.

#### Algorithm comparison in deterministic environment

First, we conducted experiments in a deterministic environment, not considering uncertain events in airport operations, as shown in Table 4. We compared the performance using FCFS (prioritizing large aircraft flights), PSO, ACO, GA, and GA_Improve (Improved Genetic Algorithm with the introduction of local evaluation factor and cooperative mutation mechanism). The comparison experiments utilized a simulation program based on Unity as a validation tool, and the simulation results were compared. To ensure the effectiveness of the experiment, all data inputs and objective weight values were set consistently.

**Table 4:** Comparison of algorithm performance.

#	Algorithm														
	FCFS			PSO			ACO			GA			GA_improve		
	obj1	obj2	wt	obj1	obj2	wt	obj1	obj2	wt	obj1	obj2	wt	obj1	obj2	wt
1	289182	543	47	268133	448	27	268654	473	35	259005	458	22	219142	446	20
2	289603	499	76	265516	441	23	276503	451	39	255918	430	21	216661	422	22
3	287671	582	56	268820	448	26	267013	449	31	254647	466	27	218077	420	20
4	282935	509	36	265518	461	20	279036	467	29	264119	461	20	208477	414	19
5	275898	583	54	265141	465	22	268703	453	36	253118	409	19	226919	430	19
6	283528	476	91	267596	441	26	263591	448	41	247098	441	23	216173	453	24
7	283842	508	68	262229	438	24	278652	449	35	260341	443	23	223361	429	22
8	286998	545	48	269984	472	22	263512	439	38	252435	473	23	211286	424	22
9	292525	492	49	269488	427	21	271003	449	31	256182	435	21	214184	410	18
10	278633	558	40	268649	448	22	269325	456	33	260021	453	20	211595	435	19
**Best**	275898	476	36	262229	427	20	263512	439	29	247098	409	19	208477	410	18
**Avg**	284381	529	56	267515	448	23	270599	453	34	255327	446	22	216266	428	20

**Figure 9:**
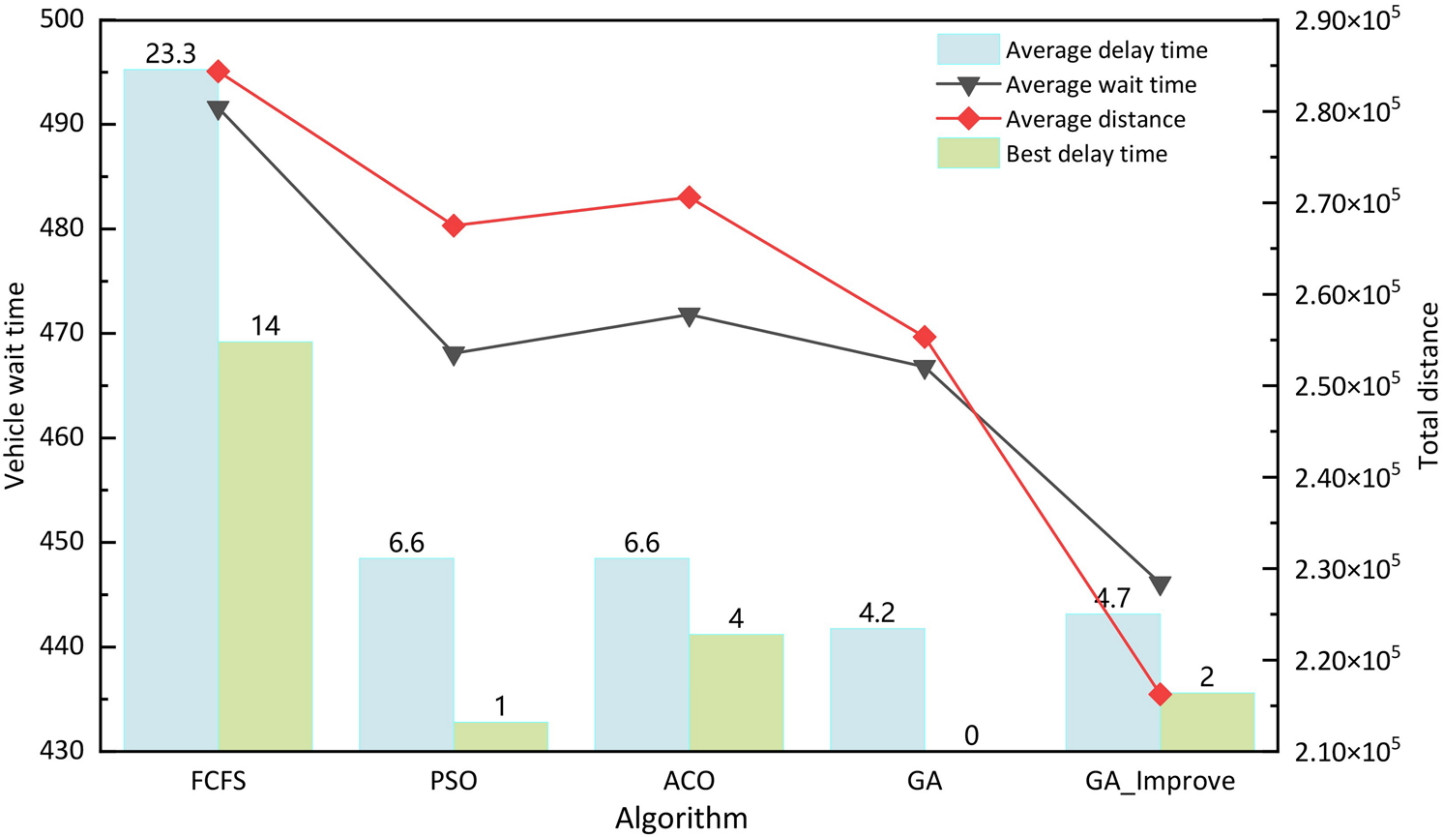
Algorithm output results comparison.

The results indicate that, after multiple simulation experiments, GA_Improve achieves the best values for both objectives (vehicle travel distance and vehicle waiting time). FCFS performs the worst, as it is a non-optimized algorithm that generates a solution in a single run without sufficient consideration of constraints and requirements across various dimensions of the solution. Particularly, FCFS fails to select the most suitable vehicles in terms of departure position and time. However, it has a short computation time, providing a solution within seconds. PSO, ACO, GA, and GA_Improve achieve better results when flight delay times are acceptable. These algorithm incorporate stricter time constraints, utilizing hard service time windows and considering more service details and constraints. As shown in Fig. 9 , GA_Improve demonstrates a significant advantage in reducing both the travel distance and waiting time during the scheduling process. Compared to the other four algorithm, GA_Improve reduces vehicle travel distances by 24.42%, 19.42%, 20.37%, and 16%, and reduces vehicle waiting times by 9.26%, 4.70%, 5.54%, and 4.43%, respectively. Efficient vehicle utilization is crucial, not only shortening vehicle travel time but also providing a buffer time for delayed vehicles, reducing the impact on the subsequent services of these vehicles.

In summary, in a deterministic environment, the scheduling model proposed in this paper and the scheduling plans generated by the GA_Improve algorithm outperform other algorithm in terms of vehicle travel distance and waiting time while ensuring low delays. Overall, this model and the improved algorithm can be effectively applied to the comprehensive scheduling of airport specialized vehicles, ensuring efficient flight services.

**Table 5:** Delay time and waiting time in different scenarios.

Algorithm	Static environment		Actual arrival and departure time		Uncertain service time		Insufficient number of vehicles		Uncertain driving routes	
	dt	wt	dt	wt	dt	wt	dt	wt	dt	wt
FCFS	14	475	82	543	43	484	77	550	42	724
PSO	1	444	57	503	33	471	41	487	21	682
ACO	4	451	64	491	29	474	46	483	25	615
GA	0	439	72	483	37	482	30	493	16	624
GA_improve	2	420	61	482	20	457	51	481	23	610

**Figure 10:**
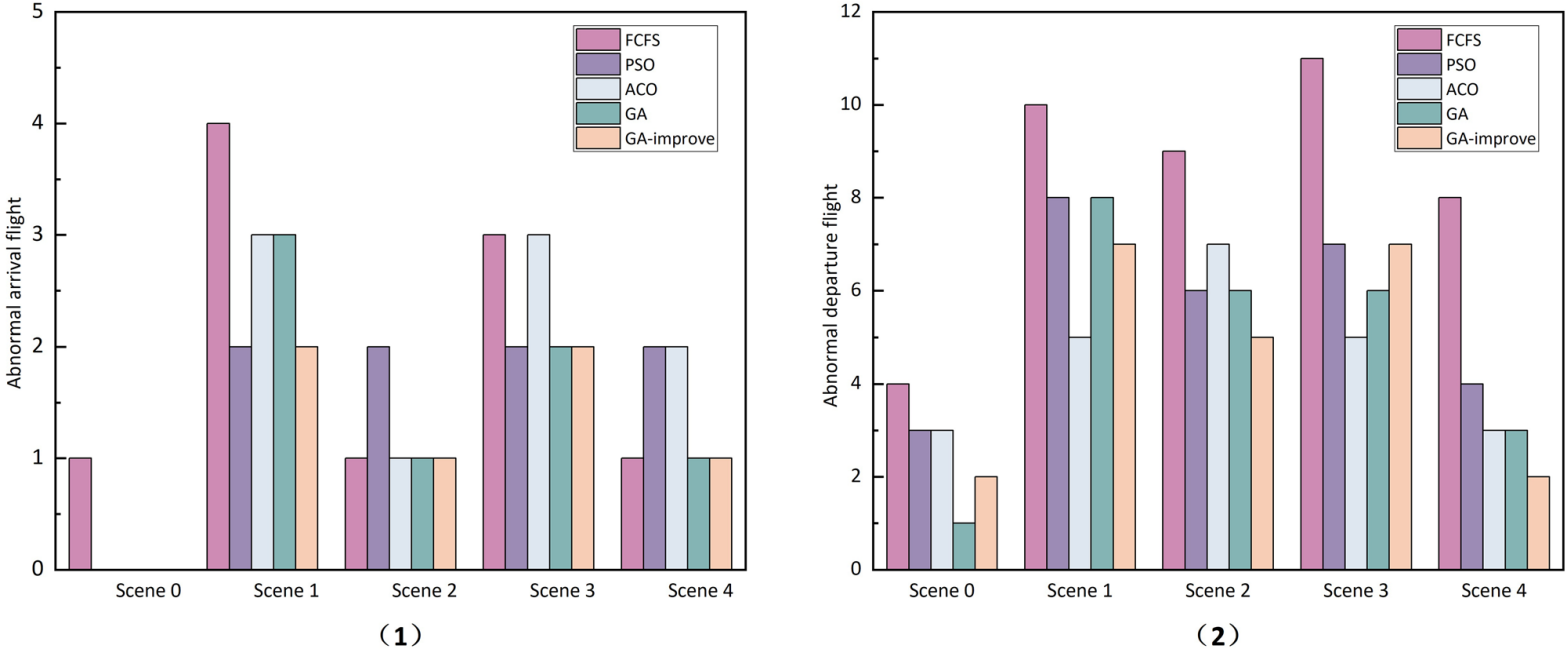
The number of abnormal flights in different scenarios: (1) Abnormal arrival flight; (2) Abnormal departure flight.

However, in airport operations, there are many uncertainties and random interferences, making it unrealistic to assume the environment is simply deterministic. To enhance the robustness of scheduling plans, these uncertainties must be thoroughly considered. For example, actual arrival and departure times of flights, vehicle service time, the available quantity of vehicles, and the selection of vehicle routes all fall under these uncertain factors. Introducing these random interferences into the simulation environment, the results indicate that all algorithmic scheduling plans designed in a deterministic environment significantly increase flight delay times and waiting service times for vehicles at the gates, as shown in Table 5. These plans are no longer applicable in a real airport environment. In an uncertain environment, the number of flights unable to take off and land on time sharply increases, especially in Scenarios 1 and 3, as depicted in Fig. 10 , where the abnormal arrival and departure flights for all five algorithm reach 14, 10, 8, 11, and 9, respectively. Currently, literature has considered uncertainty factors in optimization models, but there are two main problems: firstly, the limited consideration of uncertainty factors in optimization models, and secondly, many studies simplify uncertainty into a single parameter using mathematical methods and probability statistics. Therefore, this study explores simulation and optimization based on digital twins to solve scheduling problems. In the simulation program, we will fully consider the uncertainties at the airport and use optimization iteration and simulation feedback to enhance the feasibility, effectiveness, and robustness of scheduling plans in a dynamic environment.

#### Evaluation of SIM-GA_improve scheduling strategy

In the simulation environment, considering the actual arrival and departure times of flights, vehicle service time, insufficient vehicle quantity, and uncertainty in vehicle routing, the SIM-GA_Improve method is employed to solve the problem. The flight delay time is similar to that in a static environment, with a significant reduction in the number of flight delays and conflicts in vehicle services falling within an acceptable range. As shown in Table 6, the SIM-GA_Improve method achieves favorable values for vehicle travel distance and waiting time. In comparison to the GA_Improve method, it exhibits a slight disadvantage in total vehicle quantity. This is attributed to the consideration of additional constraint conditions in this method, and fluctuations in fitness values due to feedback from the simulation environment. Overall, in the context of airport vehicle scheduling problems, the primary objective remains to reduce flight delay time and ensure timely service for vehicles.

**Table 6:** Comparison of algorithm performance.

Algorithm	Object function		Abnormal departure flights		Abnormal arrival flights		Other Metrics		
	Obj1	Obj2	Number	Percentage	Number	Percentage	Delay time	Total Vehicles	Service conflict
GA	252435.3	552	3	0.2	9	0.36	81	98	23
GA_Improve	218991.9	480	3	0.2	5	0.2	62	88	27
SIM-GA_Improve	211545.1	456	0	0	2	0.08	8	96	6

**Figure 11:**
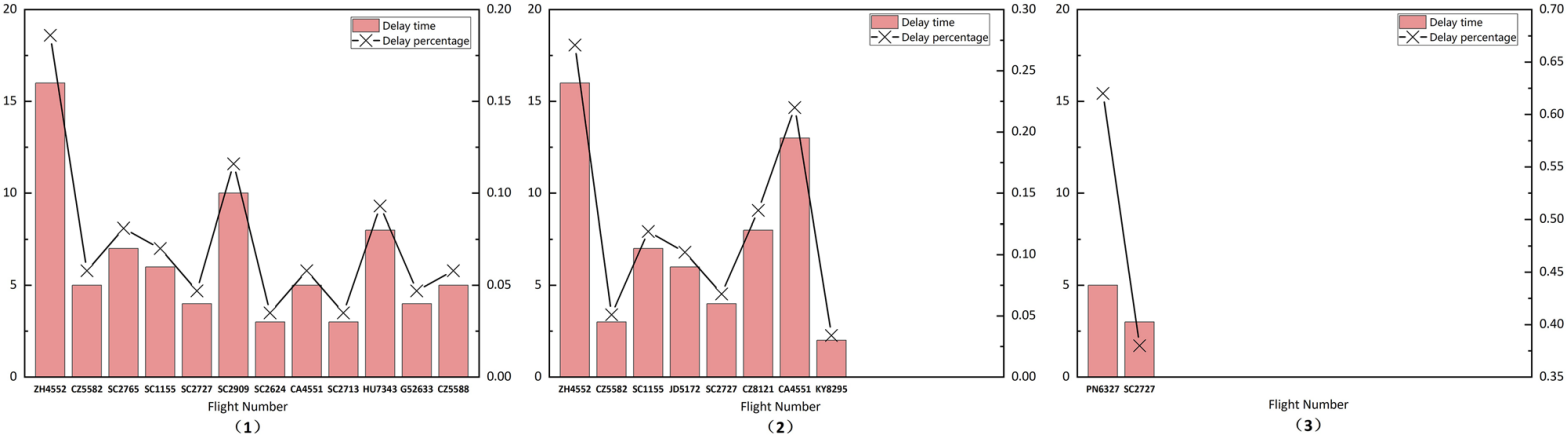
Flight delay information: (1) GA; (2) GA_Improve; (3) SIM-GA_Improve.

In the scheduling solutions generated using the GA and GA_Improve methods, as depicted in Fig. 11(1) , the GA method incurs 3 and 9 delays for arrival and departure flights, respectively. In Fig. 11(2) , the GA_Improve method results in 3 delays for arrival flights and 5 for departure flights. The delay proportions for arrival and departure flights are approximately equal for both methods, with delays primarily occurring in departure flights. As shown in Table 6, the proportions of delays for departure flights in the two methods are 0.36 and 0.2, respectively. However, the GA method exhibits a more balanced distribution of delay times.Utilizing the SIM-GA_Improve method, the overall delay time in the scheduling solution is only 8 minutes, with delays occurring for only 2 flights, and all delay times falling within the normal range, as illustrated in Fig. 11(3) . In summary, the scheduling plan obtained with SIM-GA_Improve can promptly provide reliable services for flights while mitigating delay times effectively.

**Figure 12:**
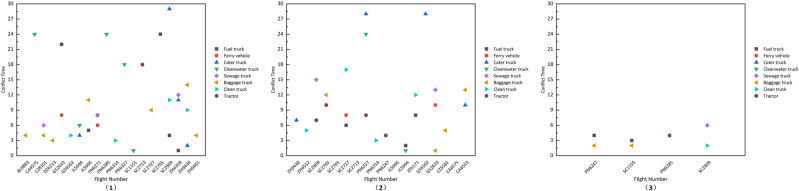
Vehicle service conflicts: (1) GA; (2) GA_Improve; (3) SIM-GA_Improve.

The relationship between flight delays and untimely service as well as service sequence conflicts with specialized vehicles is significant. Service sequence conflict refers to instances in the scheduling plan where the completion time of a vehicle’s previous service exceeds the starting time of its current service. Due to the utilization of hard time windows in the optimization model, the starting time for vehicle services falls within the window. However, due to uncertainties in service duration, travel distance, and travel time, the completion time of vehicle services may be delayed, leading to conflicts in the service sequence. Therefore, service sequence conflicts are a crucial factor contributing to flight delays. Both the GA and GA_Improve scheduling solutions exhibit a substantial number of service sequence conflicts, as illustrated in Fig. 12(1) and (2). Specifically, 31 vehicles and 25 vehicles experience service conflicts in each method, involving all eight types of specialized vehicles participating in the service. This affects 20 and 18 flights, respectively. In contrast, SIM-GA_Improve effectively mitigates a significant number of conflicts, reducing the quantity of service conflicts and average conflict time to 6 and 9 minutes, as depicted in Fig. 12(3) . This visually demonstrates the efficacy of the SIM-GA_Improve method in minimizing service conflicts.

**Figure 13:**
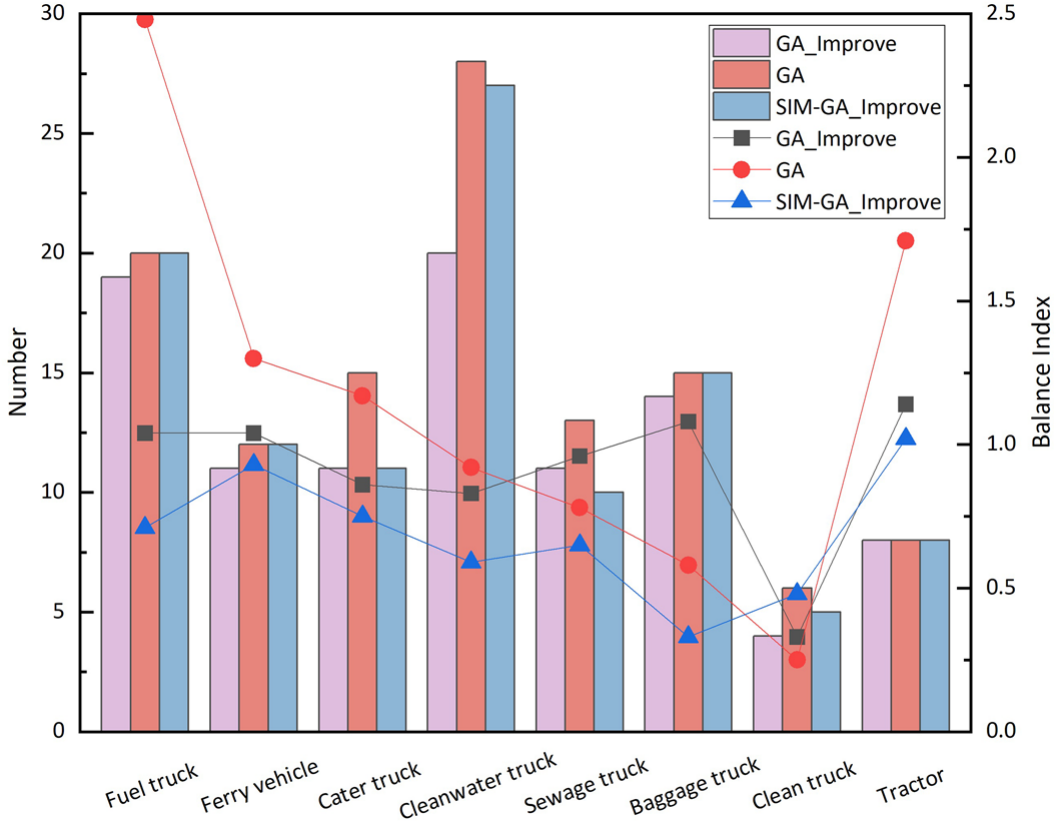
The balanced utilization of vehicles.

The balanced utilization of vehicles is also a manifestation of the maximum value that specialized vehicles can bring, as shown in Fig. 13. The number of vehicle used in the GA_Improve and SIM-GA_Improve methods is slightly less than that in the GA algorithm. Notably, SIM-GA_Improve exhibits superior balanced utilization of vehicles compared to the other two methods, optimizing the frequency of vehicle use and achieving a balance in the utilization of each vehicle.

#### Framework comparison

To compare the efficiency of our proposed framework, we compared it with the algorithms (FCFS), simulation (SIM), simulation-optimization frameworks (SSIM, SIM-FEED), and our framework DT-SIM-GA_Improve. We adopted a comprehensive metric, OTQ (optimal time and quality), to analyze the feasibility of our framework. Due to the high demand for real-time performance in DT, the computational time of the solution is crucial for scheduling. However, the quality of the solution cannot be ignored. As shown in the Table 7, solving the instances in this paper, the computational time of the framework algorithms FCFS and SIM is less because they do not require iterative calculations. However, the quality of their solutions is poor. In terms of solution quality, the objective values of SSIM, SIM-FEED, and DT-SIM-GA_Improve are acceptable. However, since all three frameworks involve feedback operations of optimization algorithms and simulation models, the overall computational time overhead is comparatively large, especially since our method utilizes a simulation model designed through Unity, requiring visualization of processes and results, resulting in the largest simulation time overhead. Fortunately, our optimization algorithm GA_Improve has strong exploration capability and stability, resulting in less computational time. From a comprehensive evaluation perspective, DT-SIM-GA_Improve better meets our scheduling requirements than simply pursuing computational efficiency and solution quality. Considering real-time performance is a crucial feature of DT, it is necessary to reduce computational time without sacrificing solution quality.

**Table 7:** Comparison of frameworks.

Framework	Algorithm time	Simulation time	Obj1	Obj2	OTQ
FCFS	8 s	0	289315	46 min	1.193
SIM	0	98 s	295647	33 min	0.947
SSIM	67 s	128 s	256871	27 min	0.815
SIM-FEED	49 s	196 s	231564	19 min	0.680
DT-SIM-GA_Improve	52 s	276 s	225986	12 min	0.597

#### Optimization of scheduling plans under emergency events

**Figure 14:**
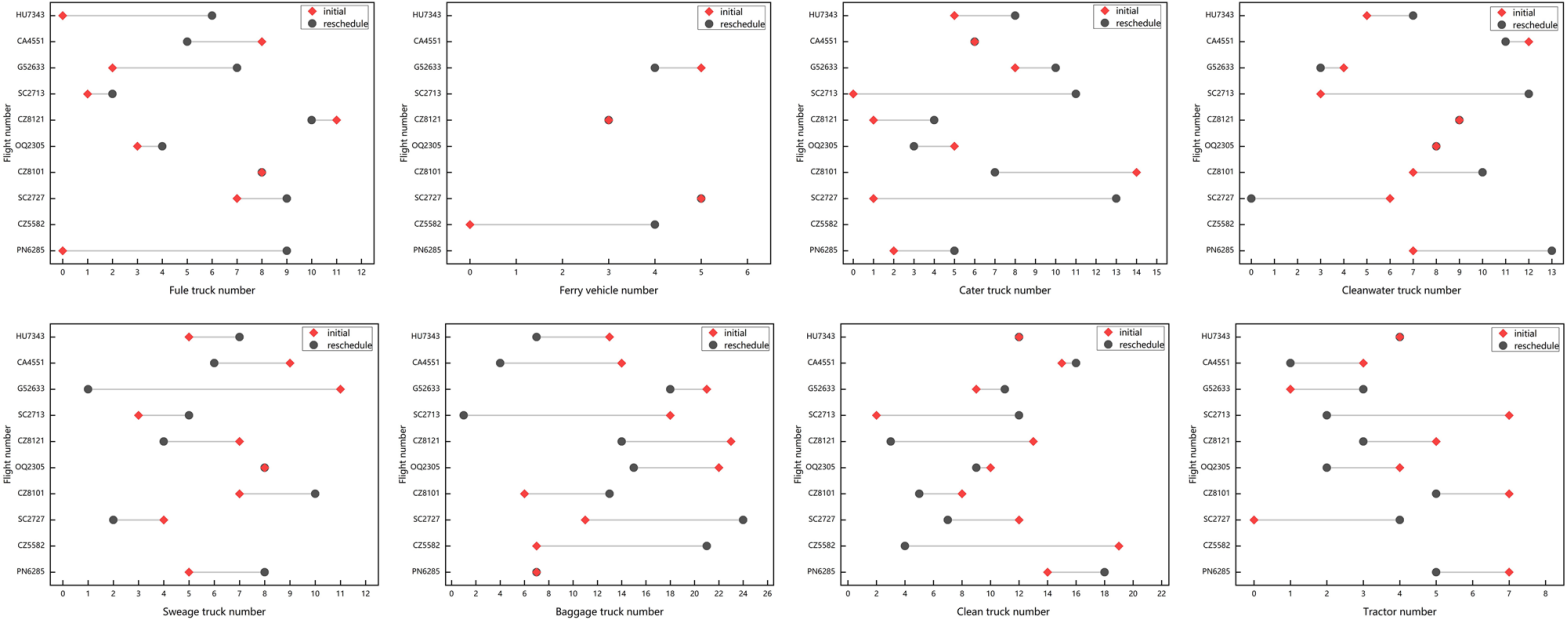
The results of the vehicle rescheduling.

If a large-scale flight delay occurs at 8 AM, the existing scheduling plan becomes obsolete, requiring a reassessment. In such a scenario, a digital twins scheduling system can promptly identify this dynamic event, collect relevant data, and trigger the reschedule algorithm at the scheduling center to generate a new scheduling plan. In this situation, all specialized vehicle services originally scheduled for execution at 8 AM or planned for execution after 8 AM will be temporarily suspended. Simultaneously, the flights involved in these services also need to be rescheduled. Overall, 15 flights are affected, with 10 facing significant delays. Figure 14 illustrates the reschedule changes for service vehicles associated with flights experiencing severe delays. When devising a new reschedule plan, considering the completion time of the last service for specialized vehicles, priority is given to scheduling available vehicles to the aircraft gates for service. This enhances the efficiency of specialized vehicle utilization, alleviating the impact of the emergency event on subsequent flight schedules.

Compared to the initial scheduling and manual scheduling, the reactive scheduling demonstrates significant advantages. As shown in Table 8, the reactive scheduling reduces the number of affected flights by 40% and 20% compared to the initial scheduling and manual schedule, respectively. Additionally, in terms of delay time and extra vehicle waiting time, the reactive scheduling also exhibits high efficiency, reducing these metrics to 51 minutes and 39 minutes. It is noteworthy that in the number of service conflicts, the initial scheduling and manual scheduling show a higher frequency of conflicts, which may lead to disruptions and inefficiencies in vehicle scheduling. The experimental results demonstrate that the digital twins-based multi-strategy scheduling framework performs exceptionally well in handling unexpected events, effectively reducing flight delay time, and enhancing the efficiency of vehicle scheduling. This provides a more effective solution for airports facing unforeseen events.

**Table 8:** Different scheduling strategies outcomes.

Scheduling strategy	Total delay time	Number of service conflicts	Number of affected flights	Percentage of affected flights percentage	Extra vehicle waiting Time
Initial scheduling	175	27	10	66.6%	93
Manual scheduling	87	17	7	46.6%	51
Reactive scheduling	36	13	4	26.6%	39

#### Futher analysis

In this section, we will conduct a further analysis of the correlation between the experimental results and the proposed algorithm and framework. Additionally, we will explore the potential challenges inherent in the framework.

Firstly, to verify that our improved genetic algorithm (GA_Improve) is more effective for solving the scheduling model presented in this paper, we tested the solution quality of five algorithms-FCFS, PSO, ACO, GA, and GA_Improve-under a deterministic environment, as shown in Table 7. GA_Improve consistently achieved better results for both objective functions over 10 rounds of validation. Additionally, it yielded better results for the auxiliary metric (total vehicle waiting time). Notably, for the first objective function (total vehicle travel distance), our method reduced the distance by 24.42%, 19.42%, 20.37%, and 16% compared to the other four algorithms, respectively. This improvement can be attributed to our enhancements in crossover and mutation operations, which increased the algorithm’s exploratory capabilities and population stability. The average optimization objectives achieved by our algorithm were superior to those of the other algorithms, demonstrating its superiority and suitability for solving the scheduling model in this paper. Next, we validated the necessity of introducing a simulation model to handle uncertainties ,such as fluctuations in flight schedules. We proposed a SIM-GA_Improve strategy to address these uncertainties. If solutions obtained using GA and GA_Improve were validated under uncertain conditions, the total vehicle travel distance and delay time would increase, with delay times exceeding acceptable limits by several times. Solutions derived from standalone algorithms would significantly deviate from actual operations in uncertain environments, making them impractical. The SIM-GA_Improve strategy effectively reduced both objective values while also decreasing the number of irregular flights and vehicle service conflicts. The improved results were due to the iterative interactions between the simulation model and the algorithm during the optimization process. The simulation model incorporated uncertainties and provided more accurate feedback to the optimization algorithm, ensuring that the algorithm converged towards the optimal solution during its evolution. When using standalone optimization algorithms, the fitness feedback values are derived from estimated objective values of the deterministic model, which cannot effectively handle uncertainties. Consequently, although the numerical results may appear satisfactory, their performance in real-world scenarios is poor. These two experiments demonstrate the superiority of our proposed GA_Improve and the effectiveness of the SIM-GA_Improve strategy in handling uncertainties. However, achieving dynamic scheduling still relies on a comprehensive framework.

We validated the performance of our proposed framework, DT-SIM-GA_Improve, against the algorithm FCFS, the simulation algorithm SIM, and the simulation-optimization frameworks SSIM and SIM-FEED, using OTQ as the evaluation metric. The results show that DT-SIM-GA_Improve balances computational time and solution quality, achieving the best result with an OTQ score of 0.579, outperforming the other frameworks. However, this achievement comes at the cost of increased computational time, as the visualization and execution of the simulation model are resource-intensive, and the interactions between the algorithm and the simulation model require additional time. Despite this, the superior solution quality validates the effectiveness of the framework. Next, to verify the framework’s capability for dynamic adjustment of solutions, we conducted experiments under scenarios of large-scale flight delays. Using the initial plan or manual rescheduling proved ineffective in handling delays, leading to continued delay propagation and exacerbated vehicle service conflicts, as shown in the Table 6. However, by employing our framework for reactive rescheduling, based on real-time data such as actual flight arrival times and real-time fleet usage, we dynamically adjusted the scheduling plan. This primarily involved rescheduling vehicles for subsequent flights affected by delays. The experimental results demonstrate that the rescheduling strategy under our framework significantly reduced delay propagation, cutting the number of affected flights by 26.6% and the additional vehicle waiting time by 39 minutes. In summary, compared to other frameworks, our framework, supported by digital twins technology, can obtain more accurate information and parameters, further improving solution quality and feasibility. In the event of sudden incidents, our framework can respond more quickly to anomalies and dynamically adjust the plan to mitigate the subsequent impacts of the incident.

For solving the initial plan, the demand for computational time is not as critical. Our framework can meet the requirements for both computational time and solution quality. However, dynamic adjustments to the plan must be completed in an extremely short time, as the digital twins has high real-time requirements. If the computation time for dynamic adjustments is too long, it will cause desynchronization between the physical and virtual spaces. Therefore, the optimization algorithm and simulation model are the two main factors limiting the responsiveness and real-time performance of the entire framework. To improve the efficiency of the framework, efforts can be made in the selection of the optimization algorithm and the design of the simulation model. Heuristic algorithms are constrained by manually designed heuristic rules and are prone to getting stuck in local optima. They lack the ability to learn actively and cannot meet the digital twin’s requirement for adjustments over time. The simulation model, visualized and verified in Unity, has high computational demands, and its design directly impacts computational efficiency. Designing rules for vehicle time steps can accelerate the entire simulation process, achieving near real-time simulation. Finally, to enable continuous operation of the framework, substantial computational resources are required, including data and model management, as well as monitoring and surveillance of the running process. This necessitates additional functional interfaces to support the continuous operation of the framework.

## Conclusion

The flight ground-handling are facing significant challenges due to numerous random interferences in airport, leading to vehicle service conflicts and low resource utilization. In this paper, we propose a multi-objective optimization model aimed at minimizing the total travel distance and vehicle waiting time, addressing the complexity and constraints of service activities in the context of the multi-type vehicle scheduling problem. Considering the advantages of genetic algorithms in global search, we design an improved genetic algorithm with a three-layer chromosome structure that incorporates enhanced crossover and mutation operations to stabilize and diversify the search process, effectively addressing vehicle routing problems with time windows. Furthermore, to achieve a more real-time and robust global solution, we introduce a DT-based multi-strategy scheduling framework for specialized vehicles. This framework integrates data from multiple sources through a data processing module and establishes an optimization module and simulation module. Through the iterative interaction between the optimization module and simulation module, we can obtain approximate optimal and reliable solutions. In the digital twins system, we monitor dynamic events at the airport and deviations between scheduling plans and the operational status of the airport. Once it is determined that the scheduling plan is no longer applicable, we use real-time data for rescheduling. The application of this framework at a major hub airport in China validated its effectiveness, demonstrating its advantages in providing efficient services and reducing delays. Moreover, with the deep integration of big data, simulation technology, and artificial intelligence, historical and real-time data at airports become more comprehensive and accurate, providing stronger technical support and data foundations for real-time vehicle scheduling.

DT-based scheduling for specialized vehicles is designed to address real-time scheduling problems, therefore, algorithm should evolve using real airport perception data. Additionally, more effective dynamic adjustment strategies should be employed when capturing dynamic events that require rescheduling. For instance,Silva introduced an adaptive scheduling algorithm^37^,Heng established dynamic programming time windows^38^, and Yao incorporated improved selection strategies and adaptive spark numbers into rescheduling algorithm to enhance the real-time responsiveness of the algorithm^27^.

## Data Availability

The datasets used and/or analysed during the current study available from the corresponding author on reasonable request.
